# Improving Crop Yield and Nutrient Use Efficiency via Biofertilization—A Global Meta-analysis

**DOI:** 10.3389/fpls.2017.02204

**Published:** 2018-01-12

**Authors:** Lukas Schütz, Andreas Gattinger, Matthias Meier, Adrian Müller, Thomas Boller, Paul Mäder, Natarajan Mathimaran

**Affiliations:** ^1^Department of Environmental Sciences-Botany, University of Basel, Basel, Switzerland; ^2^Department of Soil Sciences, Research Institute of Organic Agriculture (FiBL), Frick, Switzerland; ^3^Department of Socio-Economic Sciences, Research Institute of Organic Agriculture (FiBL), Frick, Switzerland; ^4^Department of Environmental Systems Science, Institute of Environmental Decisions, ETH Zurich, Zurich, Switzerland

**Keywords:** meta-analysis, biofertilizer, microbial inoculants, agricultural productivity, nitrogen use efficiency, phosphorus use efficiency, arbuscular mycorrhizal fungi, PGPR

## Abstract

The application of microbial inoculants (biofertilizers) is a promising technology for future sustainable farming systems in view of rapidly decreasing phosphorus stocks and the need to more efficiently use available nitrogen (N). Various microbial taxa are currently used as biofertilizers, based on their capacity to access nutrients from fertilizers and soil stocks, to fix atmospheric nitrogen, to improve water uptake or to act as biocontrol agents. Despite the existence of a considerable knowledge on effects of specific taxa of biofertilizers, a comprehensive quantitative assessment of the performance of biofertilizers with different traits such as phosphorus solubilization and N fixation applied to various crops at a global scale is missing. We conducted a meta-analysis to quantify benefits of biofertilizers in terms of yield increase, nitrogen and phosphorus use efficiency, based on 171 peer reviewed publications that met eligibility criteria. Major findings are: (i) the superiority of biofertilizer performance in dry climates over other climatic regions (yield response: dry climate +20.0 ± 1.7%, tropical climate +14.9 ± 1.2%, oceanic climate +10.0 ± 3.7%, continental climate +8.5 ± 2.4%); (ii) meta-regression analyses revealed that yield response due to biofertilizer application was generally small at low soil P levels; efficacy increased along higher soil P levels in the order arbuscular mycorrhizal fungi (AMF), P solubilizers, and N fixers; (iii) meta-regressions showed that the success of inoculation with AMF was greater at low organic matter content and at neutral pH. Our comprehensive analysis provides a basis and guidance for proper choice and application of biofertilizers.

## Introduction

The current alarming rate of decline of earth's natural resources, particularly of the reserves of rock phosphate and fossil fuel, is of great concern for the future of agriculture, particularly in developing countries (St.Clair and Lynch, 2010). Not surprisingly, sustainable crop production remains a major global challenge and has drawn increasing attention among policy makers, business, and the scientific community (Seufert et al., 2012; Wezel et al., 2014). Efforts to mitigate the declining mineral nutrient reserves are currently major topics of research but the perturbance of the global biogeochemical cycles, mainly driven by the use of mineral fertilizers, remains a serious problem (Kahiluoto et al., 2014).

Microbial inoculants, so-called biofertilizers, are a promising technology to reduce the use of conventional inorganic fertilizers. Many of them can serve as biofertilizers as they are able to fix nitrogen (N), help to access nutrients such as phosphorus (P) and N from organic fertilizers and soil stocks, improve drought tolerance, improve plant health or increase salt tolerance (Vessey, 2003; Arora, 2013). The effects of biofertilizer applications have often been inconsistent, hindering their widespread adoption by farmers. The reasons can be manifold, such as soil conditions, strain identity, or host genotype. Yet, the long history of research offers a great reservoir to identify key influencing factors. Numerous reviews on microbial inoculants have been published, but quantitative results are scarce. For example, McGonigle (1988), Lekberg and Koide (2005), and Berruti et al. (2016) analyzed the potential of AMF (arbuscular mycorrhizal fungi) as biofertilizers. Rubin et al. (2017) studied the influence of PGPR (plant growth-promoting rhizobacteria) especially under drought conditions. Nevertheless, what is missing is a comprehensive quantitative analysis over all biofertilizers and across all target crops and climatic conditions at global scale.

Here, we conducted a quantitative evaluation of the pertinent literature in the form of a meta-analysis. Its objective was to quantify the effect of biofertilizers on the performance indicators crop yield and P and N nutrient use efficiencies.

The following hypotheses were addressed: (i) across all studies, biofertilizer show a significant positive effect on crop yield and nutrient use efficiency; (ii) there is a difference in biofertilizer response between categories of crops; (iii) climate is a major factor for the constituency of soil biodiversity, soil fertility and soil carbon content, and thus the performance of biofertilizers; (iv) P availability is a limiting factor in many soils. P levels are expected to influence activity and thus effectivity of biofertilizers. Especially phosphate-solubilizing bacteria and AMF are expected to be affected by P levels.

## Materials and methods

### Search strategy

Peer reviewed publications (and the reference lists from these publications) were searched for between May 2015 and February 2016 in Web of Science by Thomson Reuter, Scopus by Elsevier and Google Scholar with the following keywords “biofertilizer OR biofertiliser OR microbial inoculants.” Only studies using data from field trials to more closely reflect real farming practices and providing separate data for each treatment and written in English language were selected. Studies were only included when they had conducted pairwise comparison between the application of a biofertilizer to a non-treated control under the same pedo-climatic conditions (e.g., temperature, precipitation, soil texture, and type), and if the biofertilizers had been tested under the same input level of inorganic and organic fertilizers as the paired non-inoculated control. Studies had to report the treatment mean of yields, its standard deviation (SD) and number of replications (*n*) to calculate the different use efficiencies and effect sizes. When fertilizer was applied the amount and type of fertilizer was required to calculate nutrient-use efficiencies for phosphorus (P) and nitrogen (N). Field trials were not included when soils were previously fumigated or heat sterilized to obtain a control without soil biota, because nutrients may be released, soil microbial community disturbed and inoculation success put at risk (Smith and Read, 2008a). If data were missing or only supplied in summarized format, authors were contacted to obtain these data. A total of 633 possible studies were identified, 222 were excluded after a first screening for greenhouse studies (except three studies with tomato grown under commercial conditions) and reviews and again 240 because they did not match eligibility criteria mentioned above (see flow diagram in Figure S1).

### Data sources

One hundred and seventy-one studies (see study list in Supplementary Data Sheet S1) proved to be eligible for our meta-analysis enabling us to generate 1,726 pairwise comparisons.

### Data preparation and descriptive statistics

All data was extracted and compiled in an excel file. If the data were only available in graph format, Plot Digitizer Version 2.6.6 (http://plotdigitizer.sourceforge.net) was used. The data was structured after biofertilizers, crops and climate. Tables 1 and **4** summarize the characteristics of crop and climate categories for the number of included studies, amount of fertilizer applied and climate representation. pH was usually given as measured in water. If pH was measured in CaCl_2_, conversion was calculated (Land Resources Management Unit, Institute for Environment and Sustainability, 2010). If the method was missing it was assumed to be measured in water. Soil pH was later used as a control variable for meta-regression.

**Table 1:** Database as related to different crop categories, climatic zones and nutrient inputs.

	**Cereals**	**Root crops**	**Legumes**	**Vegetables**	**Other crops**
Number of studies^*^	86	8	38	17	28
Number of pairwise comparisons^**^	681	137	521	142	184
Coverage of climatic zones (after Koeppen)	Aw, BSh, BSk, BWh, Cwa, Cfa, Csa, Cfb, Cwb, Dsb, Dsa, Dfb, Dwb	Aw, Cfb, Cwa, Csa, Dfb	Aw, BSk, BSh, BWh, Cwa, Cwc, Csa, Cwb, Dwa, Dsb, Dsa,	Aw, BWh, Csa, Cwa, Cfa, Cwb, Dfb,	Am, Aw, BSk, BWh, BSh, Cwa, Cwb, Csa Dsb,
Coverage of continents	5	3	4	4	3
Average N applied (kg ha^−1^) (mean/median ±*SD*)	100.7/80.0 ± 84.6	127.2/102.5 ± 75.4	44.2/22.5 ± 56.6	159.2/200.0 ± 63.0	158.2/110.0 ± 214.7
Average P applied (kg ha^−1^) (mean/median ±*SD*)	50.3/40.0 ± 37.2	56.3/52.4 ± 35.6	33.0/25.0 ± 24.8	53.1/53.7 ± 19.1	56.4/40.0 ± 56.2
Percent unfertilized of pairwise comparisons	27.90	0	19.77	9.86	8.15

Averages for fertilizer applications were only calculated if fertilizer were applied;

* Some studies appear in more than one crop category resulting in a higher (177) sum of studies than reported (171);

** *Legumes comparison with rhizobia as control (61 comparisons from 12 studies) are not included in the category for legumes resulting in lower (1665) sum of comparison than the reported (1726)*.

Bulk density was only available for 10 studies. For the others bulk density was estimated with the pedo-transfer function (Post and Kwon, 2000). Bulk density was necessary to convert soil available P from mg/kg to kg/ha. Soil available phosphorus was calculated to a depth of 30 cm. Soil available phosphorus was measured mostly with the method by Olsen, but also with Bray, Mehlich, and AB DTPA. Yet in many cases the method was not given. Yli-halla (2016) state that usually there is a rough agreement between the results obtained with different extraction methods in non-calcareous soils, but in calcareous soils the results of acidic and basic extractants usually have a poor correlation. Hence the values of soil available phosphorus cannot be seen as absolute values but only as an indicator for the real values. Soil available phosphorus was calculated to provide another perspective on phosphorus other than P use efficiency (PUE). Since no formula exists to account for available phosphorus from soil and fertilizer we conducted a meta-regression with the sum of soil available P and fertilizer P. Thus, for a comprehensive picture, we provide three different analyses of functional biofertilizer categories to P.

### Meta-analysis

A random-effects model was chosen as the statistical model for the meta-analysis (Viechtbauer, 2010b). In a meta-analysis, ideally, independent estimates should be aggregated (Borenstein et al., 2009), but in reality, and also in this meta-analysis this cannot be fully assured. Independence is violated in the cases, where several treatments are compared to the same control. It is likely also violated for the cases where study results over several years from the same comparison plots were not averaged but included separately in the meta-analysis. In both cases, we retained all data because the aim of the meta-analysis was to include as much information as possible. For the second case, N use efficiency (NUE) and P use efficiency (PUE) likely depend strongly on the annually different climate conditions, thus rather mitigating dependence. If values were supplied as an average over years, replicate numbers of each year were multiplied by the number of years. The random-effects model assumes that the single effect size depends on the study context and that studies differ in their methods and sample characteristics. As a result, there are different effect sizes among all studies. Since the true effect size and its variance are not known the restricted maximum-likelihood estimator (REML) was used (Viechtbauer, 2010b). Outliers were identified via DFBETAS values inside the R package “metafor” (Viechtbauer, 2010a).

### Effect sizes and their modeling

Effect sizes indicate the magnitude of the effect of the improved practice over the control practice concerning yield responses and nutrient use efficiency (Borenstein et al., 2009). In this study, the percent increase in dry matter yields was used for comparing yields and raw mean difference was used as effect size measure for PUE and NUE, calculated as the log transformed ratio of the mean.

### Performance indicators

In this study, we evaluated quantitatively the effects of all categories of biofertilizers on crop yield, PUE and NUE, with a main focus on relative crop yield. Key characteristics of the studies can be found in the Supplementary Data Sheet S2. Yield is defined as harvested dry main product, in form of grains, fruits, tubers or shoots. Dry weight had to be calculated for most studies. If the water content was not available, values were taken from Church and Bowes (Church and Bowes, 1966). PUE was calculated as the yield increase of dry main product per unit of P fertilizer input, and NUE accordingly as the yield increase per unit N fertilizer input referring to the agronomic efficiency of P and N, respectively (Ladha et al., 2005).

The following formulae were used:

(1) $$\begin{array}{l} Yield\;response\;\left(\%\right)=\frac{{Yield\;}_{inoculated}\;\times 100}{\;Yield_{non-inoculated}} \end{array}$$

(2) $$\begin{array}{l} \Delta PUE={\frac{Yield\;\left(kg\;ha^{-1}\right)}{Fertilizer\;P\left(kg\;ha^{-1}\right)}}_{inoculated}\\ -\;{\frac{Yield\;\left(kg\;ha^{-1}\right)}{Fertilizer\;P\left(kg\;ha^{-1}\right)}}_{non-inoculated} \end{array}$$

(3) $$\begin{array}{l} \Delta NUE={\frac{Yield\;\left(kg\;ha^{-1}\right)}{Fertilizer\;N\left(kg\;ha^{-1}\right)}}_{inoculated}\\ -\;{\frac{Yield\;\left(kg\;ha^{-1}\right)}{Fertilizer\;N\left(kg\;ha^{-1}\right)}}_{non-inoculated} \end{array}$$

Given the lack of data for estimating or modeling these additional N sources and P, the chosen approach to calculate PUE and NUE is most adequate. Nevertheless, it may lead to different effects regarding soils and nutrient loss to the environment. In case higher PUE or NUE are observed with biofertilizers with identical P and N fertilizer inputs, the biofertilizer must have resulted either in more efficient uptake of those inputs, or in making additional inputs from the soil pool available. In the first case, nutrient mining effects of soils is unlikely and potential runoff is reduced; in the second case, some nutrient mining may occur, if runoff is not reduced, e.g., if nutrients mobilized from the soil and taken up by the plant are replaced in the soil by nutrients from the fertilizer input. With the available data, we cannot discern these two cases. We report yield response in percent thereby neglecting the actual values and their size. Percentage values are necessary to normalize the yields. But percentage values are insensitive to whether the yields are already at a maximum or whether there are yield gaps in terms of other management techniques which pose a different potential to decrease or increase yields by the inoculated biofertilizers. The calculation follows the general methods used by Batten (1992). Due to lack of information on the soil types of the studies, which are crucial for the absorption of phosphorus, we believe that this method reflects PUE the best. NUE was calculated as yield of dry product by N fertilizer input. This calculation is widely used for studies in an agricultural context and referred to as agronomic nitrogen use efficiency (Yadav, 2003; Ahmad et al., 2009; Zhang et al., 2016). Yet it is criticized because it does not reflect N inputs from atmospheric deposition, nitrogen fixation and mineralization from organically bound nitrogen (Godinot et al., 2014). These inputs were not reported and are difficult to model. Our calculation is thus an apparent nitrogen use efficiency and needs to be looked at as an indicator for total nitrogen use efficiency.

### Crop and biofertilizer categories

Data were grouped into the main crop categories cereals, root crops, legumes, and vegetables. Spices like fennel or anise, cotton and oil crops were classified as other crops (see Table 2). To structure the effects of the microbial inoculants, they were classified for their P solubilization and N fixation activity. In this way, it was also possible to account for combined inoculation with different inoculants. The information on the main traits of the inoculants was taken from the studies and further literature sources. Thus, five categories were distinguished: Arbuscular mycorrhizal fungi, P solubilizers, N fixers, a combination of both P solubilization and N fixation, either in one strain or by applying two strains, and other biofertilizers with unspecified modes of action, also in combination with AMF (see Table 3). It allowed to classify biofertilizers according to their needs of phosphorus by relating their effect to plant available P in soil, thus providing direct guidance to practitioners and farmers at which level which biofertilizer is most promising.

**Table 2:** Crops included in this meta-analysis.

**Crop category**	**Crops included**
Cereals	Barley, durum wheat, rice, spring wheat, winter wheat, pearl millet, maize, sorghum, kamut, silage maize, ryegrass, finger millet
Legumes	Blackgram, chickpea, peanut, horsegram, kidney bean, mung bean, fenugreek, lentil, snap bean, soybean, runner bean, pigeon pea
Root crops	Garlic, potato, turmeric, sugar beet, cassava
Vegetables	Eggplant, tomato, cabbage, watermelon, pepper, okra, cucumber, melon
Other crops	Dill, anise, rapeseed, cotton, sesame, fennel, coriander, sunflower, mustard, sugarcane

**Table 3:** Categorization of microbial inoculants according to species characteristics and functionality.

**Category**	**Species**
AMF	*Entrophosphora colombiana, Glomus caledonium, G. clarum, G. etunicatum, G. fasciculatum, G. hoi, G. intraradices* (new name: *Rhizophagus irregularis*), *G. mosseae, Gigaspora rosea*
P solubilizers	*Arthrobacter chlorophenolicus, Bacillus firmus, B. megaterium, B. mucilaginous, Burkholderia caryophylli, Enterobacter asburiae, Microbacterium arborescens, Paenibacillus* sp., *P. polymixa, Penicillium bilaii, Providencia* sp.*, Pseudomonas aeruginosa, P. argentinensis, P. cepacia, P. chlororaphis* subsp. *aurantiaca, P. diminuta, P. fluorescens, P. fragi, P. jesseni, P. marginalis, P. paleroniana, P. putida, P. striata, P. syringae, P. tolasii, Serratia marcescens, Staphylococcus saprophyticus*
N fixers	*Anabaena azollae, A. cylindrica, A. oscillaroides, A. variabilis, A. torulosa, Aphanothece* spp., *Aulosira fertilissima, Azolla caroliniana, Azospirillum brasilense, A. lipoferum, Azotobacter brasilense, A. chrooccocum, Bacillus polymyxa, B. subtilis, Beijerinckia indica, Bradyrhizobium diazoefficiens, B. japonicum, Brevundimonas diminuta, Burkholderia vietnamensis, Calothrix* sp.*, C. elenkinii, Gloeotrichia* sp., *Gluconacetobacter diazotrophicus, Herbaspirillum seropedicae, Klebsiella pneumoniae, Mesorhizobium ciceri, Nostoc muscorum, N*. sp.*, Rhizobium leguminosarum, Staphylococcus* sp., *Tolypothrix tenuis*
N fixers plus P solubilizers	Strains of *Bacillus megaterium, B. polymixa, Enterobacter* sp., joint inoculations of P solubilizers and N fixers
Other biofertilizers	*Actinomycetes, Aspergillus niger, A. tubingensis, Bacillus circulans, B. mycoides, B. pummilus, B. simplex, B. subtilis, Burkholderia tropica, Citrobacter freundii, Kurthia* sp., *Ochrobactrum anthropic, O. ciceri, Penicillium brevicompactum, P. solitum, Piriformopora indica, Rhodobacter capsulatus, Rhodopseudomonas* sp., *Rhodotorula glutinis, Thiobacillus* sp.*, T. thioxidans, Trichoderma atroviride, T. harzianum, Variovorax paradoxus*, joint inoculations with AMF

### Climate classification and other site characteristics

The study locations were classified according to an updated Köppen climate classification (Peel et al., 2007). Thereby the studies were split into dry (BSh, BSk, BWh, Csa) and tropical climate (Aw, Am, Cwa, Cwb, Cwc, Cfa,), continental climate (Dfb, Dsa, Dwa, Dwb, Dsb), and oceanic climate (Cfb). In many studies, the experiments were performed under irrigated conditions or planted in the rainy season. Thus the climate classification is often rather an indicator for potential soil fertility and related indicators such as soil carbon than climate itself (Table 4). Because regions with Mediterranean climate have low soil carbon contents they were grouped into dry climate as well. This grouping enabled us to make a cross comparison of different biofertilizer categories and to identify key conditions for the successful application of biofertilizers.

**Table 4:** Database as related to climatic zones and nutrient inputs.

	**Tropical climate**	**Dry climate**	**Continental climate**	**Oceanic climate**
Nr of studies^a^	70	71	17	8
Nr of pairwise comparisons	686	718	152	110
Coverage of continents	5	5	3	3
Average N applied (kg ha^−1^) (mean/median ± SD)	90.8/60 ± 88.2	120.5/90 ± 132.1	78.2/80 ± 58.3	65.3/47.5 ± 45.4
Average P applied (kg ha^−1^) (mean/median ± SD)	47.3/38 ± 35.1	48.6/35.7 ± 40.7	37.8/34.9± 29.4	55.0/70.0 ± 30.2
Average OM% (mean/median ± SD)	1.69/0.88 ± 1.59	1.02/0.95 ± 0.79	2.37/1.8 ± 1.85	4.82/4.18 ± 2.85
Average pH (mean/median ± SD)	6.66/6.80 ± 1.20	7.81/7.80 ±0.34	7.16/7.15 ± 0.61	5.55/5.50 ± 0.98

*Averages for fertilizer applications were only calculated if fertilizers were applied*.

a *Five of the studies analyzed were excluded because they could not be assigned unequivocally to one climate zone*.

### Data analysis

The dataset used for this study is available in the Supplementary Data Sheet S2. The meta-analysis was conducted with R Software Version 3.2.3 and the interface R-Studio Version 0.99.491 using the “metafor” package (Viechtbauer, 2010b). Also the meta-regressions were calculated within this package by designating moderator variables which were used to calculate a mixed effects model (**Figures 6**–**8**). Selection bias was assessed with funnel plots (Figure S2) and outlier analysis was undertaken via DFBETAS values inside the R package “metafor” (Viechtbauer, 2010b).

### Missing values

Sometimes the nutrient content of organic fertilizers was not available, values were then taken from a booklet within a national project on organic farming by the Indian government (Chandra, 2005). Where bulk density was missing, it was estimated with the pedo-transfer function by Post and Kwon (2000):

(4) $$\begin{array}{l} \text{BD}=\frac{100}{\begin{array}{c} \left(\frac{OM_{conc}}{0.244}\right)+\left(\frac{100-OM_{conc}}{1.64}\right) \end{array}} \end{array}$$

where 0.244 is the bulk density of organic matter, 1.64 the bulk density of soil mineral matter, and OM_conc_ the concentration of soil organic matter (%), which was estimated according to Nelson and Sommer (1982), if necessary:

(5) $$\begin{array}{l} OM_{conc}=1.72\times SOC_{conc} \end{array}$$

Missing errors were estimated from the average reported standard deviations in percent, differentiated per crop groups. For cereals, the standard deviation (SD) was 15.2%, for legumes SD 5.5%, for melon and water melon SD 35.9%, for vegetables SD 11.2%. For maize (SD 10.6%), cotton (SD 14.0%), rice (SD 14.18%), mustard and rapeseed (SD 10.2%) values were averaged within each type of crop. Average of all were applied for anise, fennel, dill, sesame, sunflower, coriander, garlic, ryegrass, turmeric, silage maize, potato, sugarcane SD 12.0%. The standard deviation in yield as a percentage was used to estimate the error in PUE and NUE.

### Bias assessment

It cannot be excluded that there was a certain publication bias within the results. In order to find out whether there was a publication bias in the meta-analysis “funnel plots” were used to detect a possible publication bias. The trim and fill method was used to help interpretation as proposed by Duval and Tweedie (2000a,b) and Duval (2005). Modest bias was found in some groupings (Figure S2), but no studies were excluded.

## Results

**Figure 1:**
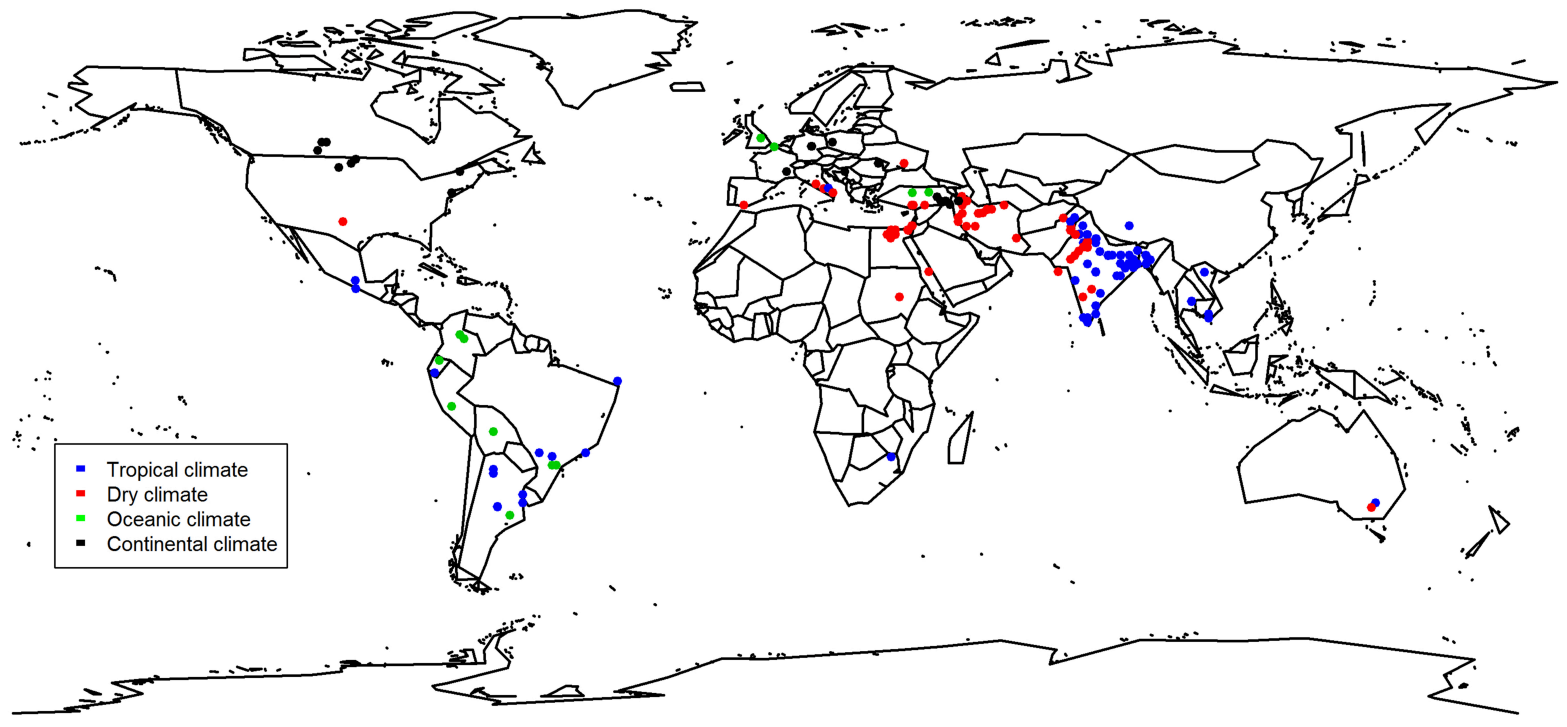
Map showing origin of the study and their classification based on the climate. Some locations were not given by the study and were thus located with the name of the place given. Studies that were conducted under commercial conditions in the greenhouse are excluded from this map (Gravel et al., 2007; Luna et al., 2012; Bernabeu et al., 2015; all tomato).

**Figure 2:**
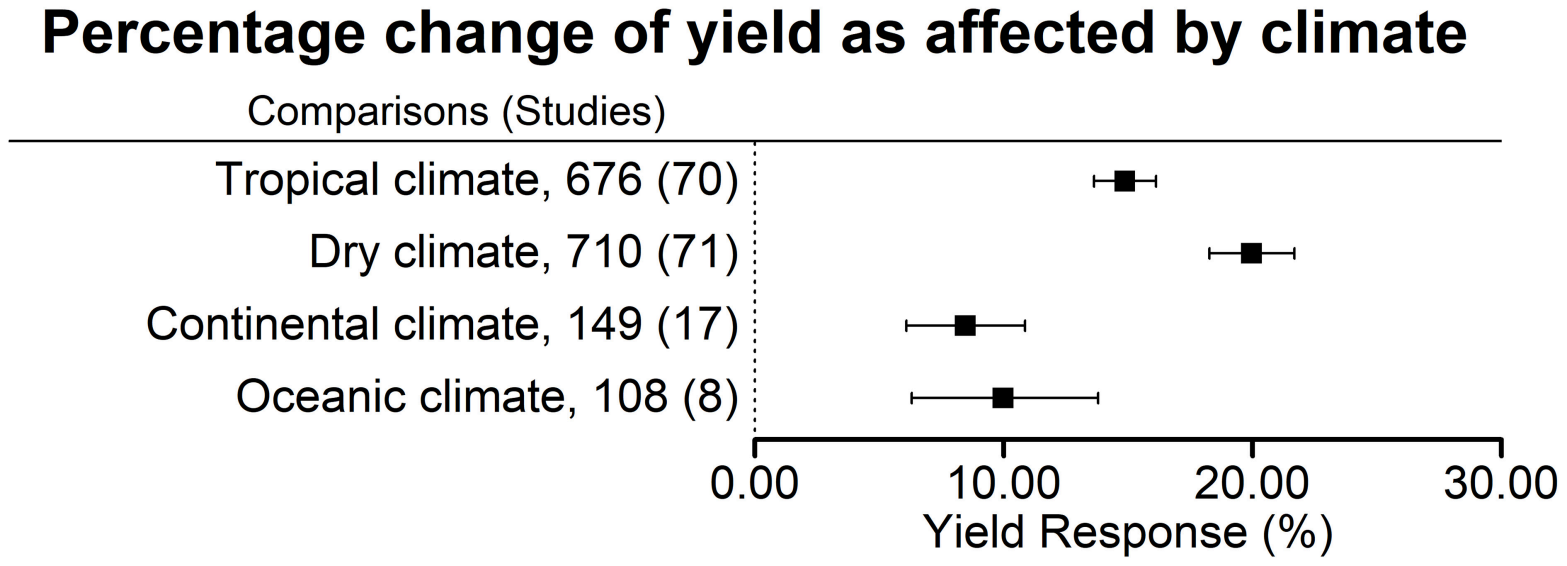
Percentage change of yield in response to biofertilizer application as affected by climate. Mean values and 95% confidence intervals of the back-transformed response ratios are shown. There was a more pronounced effect in tropical and dry climates.

**Figure 3:**
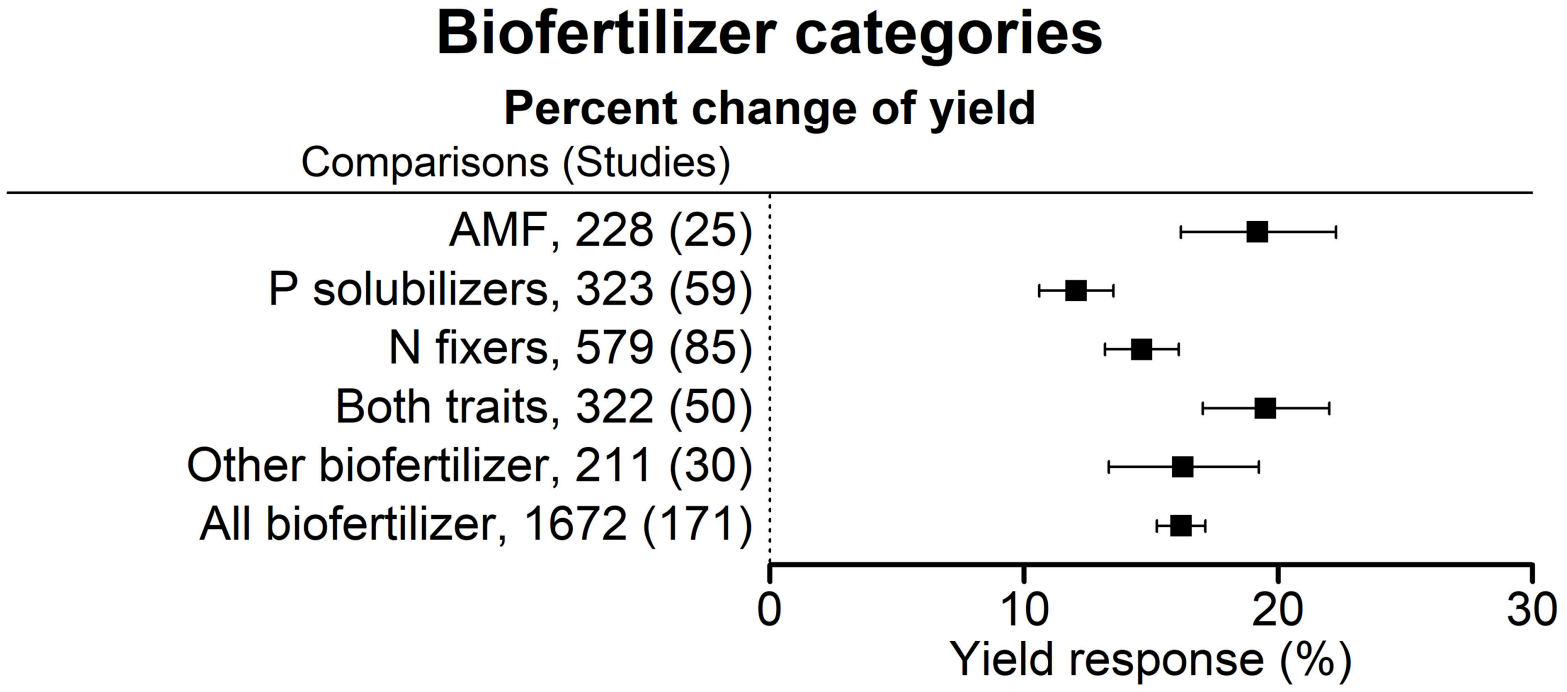
Percentage change of yield in response to the application of various categories of biofertilizers. Mean values and 95% confidence intervals of the back-transformed response ratios are shown. There was a more pronounced effect with AMF and for N fixers in combination with P solubilizers.

Our comprehensive meta-analysis with studies from all over the world (Figure 1) revealed that biofertilizers were found to be most effective in dry climates (Figure 2). Biofertilizer also improved PUE and NUE greatly. Furthermore, we found that biofertilizers possessing both N fixing and P solubilizing traits have the highest potential to improve the crop yields (Figure 3). Interestingly, AMFs, known for facilitating P nutrient uptake in plants, were on par with applications of biofertilizers with the combined traits of N fixation and P solubilization, indicating the big potential of AMFs as sole biofertilizer for most crops and climatic situations.

### Yield impact of biofertilizers by climate

Averaged across all biofertilizer categories, yield was increased the most in dry climates (+20.0 ± 1.7%), followed by tropical climates (+14.9 ± 1.2%), oceanic climates (+10.0 ± 3.7%), and continental climates (+8.5 ± 2.4%) (Figure 2). For interpretation, it is important to keep in mind that 45% of the comparisons in dry climate were conducted in the presence of irrigation. In a separate analysis of the data from dry climates, we found a significant difference in the yield increase under irrigated conditions with +15.9 ± 2.0% (316 comparisons, 39 studies) and under rainfed conditions with +21.0 ± 3.1% (274 comparisons, 20 studies). In dry climates soils had the highest pH and the lowest soil organic matter (OM) content; here, the highest amount of N fertilization was used (Table 4). However, in all climates, the variation of fertilizer application levels within the trials was high.

### Yield impact of different biofertilizer categories

AMF, other biofertilizers and the application of biofertilizers with both functional traits—N fixation and P solubilization—were the most effective inoculants. The combination of both functional traits was more effective than the separate application of biofertilizers with one trait only (Figure 3).

### Impact of biofertilizers by crop categories

**Figure 4:**
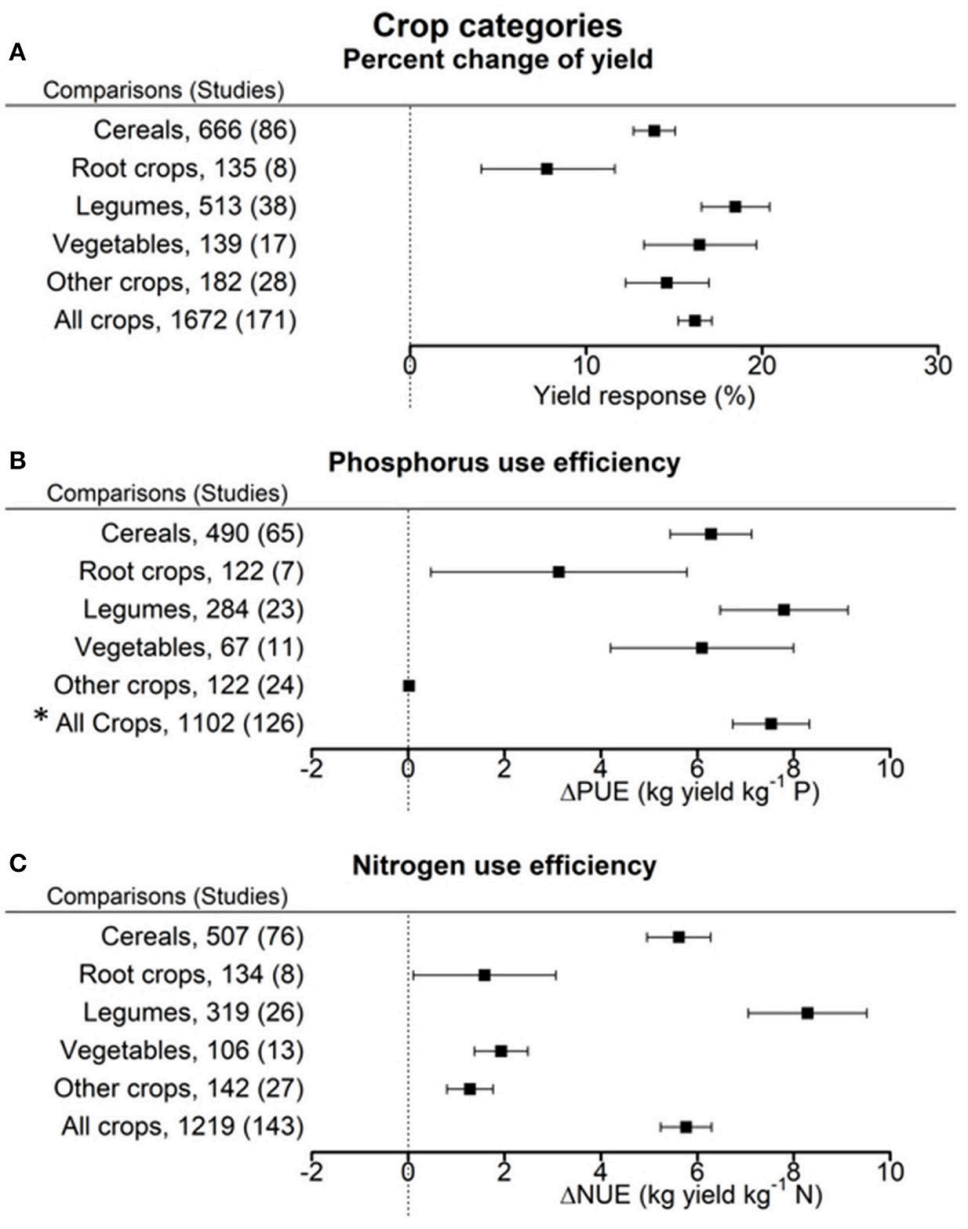
Percentage change of yield **(A)**, change in phosphorus use efficiency (PUE) **(B)**, and nitrogen use efficiency (NUE) **(C)** in response to biofertilizer application. Mean values and 95% confidence intervals of the back-transformed response ratios are shown. Yields of root crops were least responsive due to inoculation. PUE was improved in legumes, cereals and vegetables. NUE was improved in legumes and cereals but only to a minor extent in root crops and the other crops. ^*^The high value for all crops is caused by the outlier calculation that resulted in different pairs being excluded for the full sample and the sub-samples.

Across all crop categories, the inoculation with biofertilizers showed an average yield increase by 16.2 ± 1.0% as compared to non-inoculated controls (Figure 4A). Yield response was distinctly lower for root crops than for all other crop categories, with legumes showing a tendency to superior response upon inoculation.

The overall improvement of PUE due to biofertilizers was 7.5 ± 0.8 kg yield per kg P (Figure 4B). PUE increase was most pronounced in legumes (7.8 ± 1.3 kg yield per kg P). Least improvement was found with root crops and the category other crops. On average NUE was improved by 5.8 ± 0.6 kg yield per kg N fertilizer through biofertilization (Figure 4C). Legumes manifested the highest response for NUE (8.3 ± 1.2 kg yield per kg N), root crops, vegetables, and the category other crops the lowest.

### Response of biofertilizers to plant available phosphorus in soil

**Figure 5:**
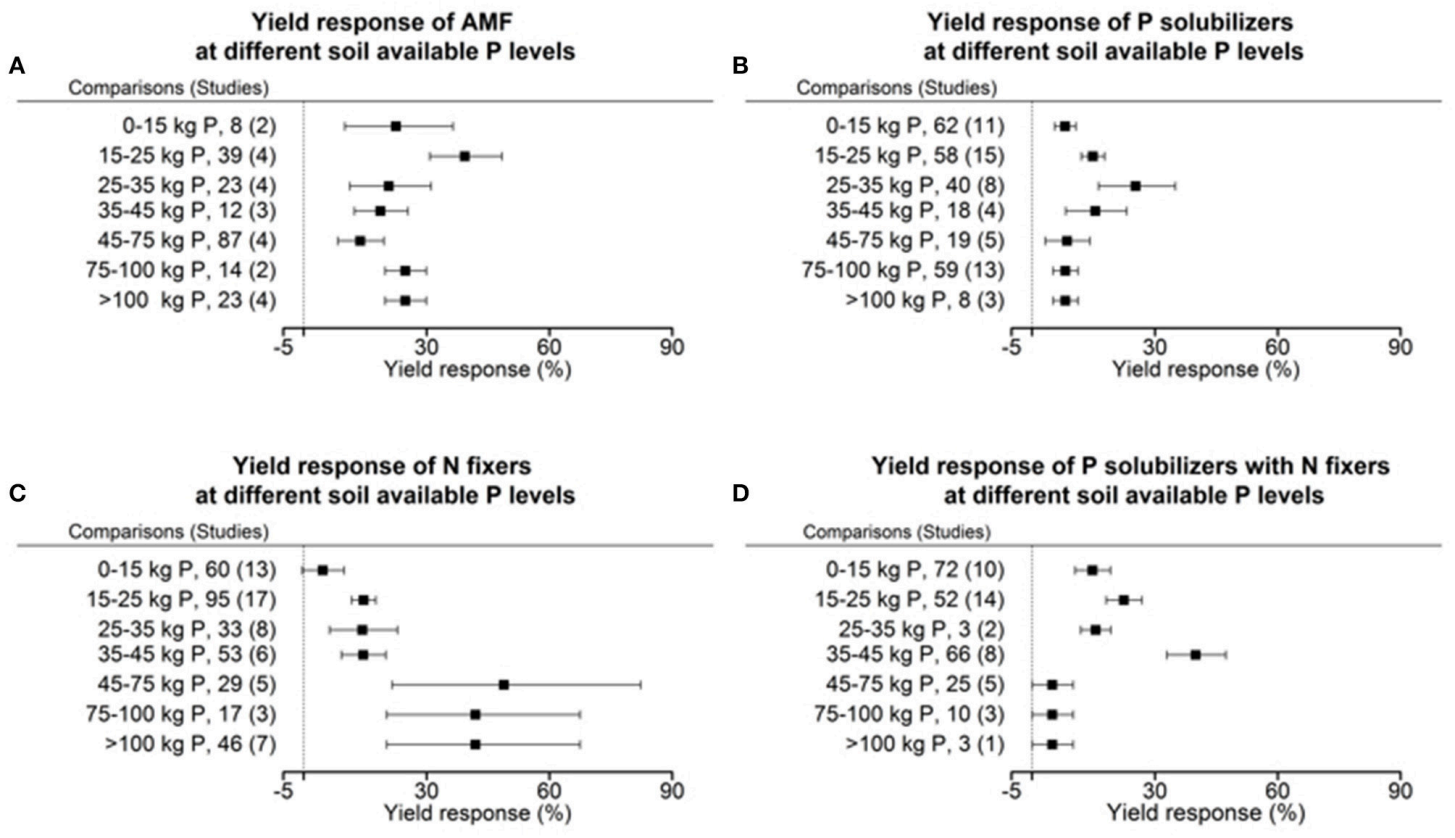
Percentage change of yield in response to applications of AMF **(A)**, P solubilizers **(B)**, N fixers **(C)**, and N fixers in combination with P solubilizers **(D)** as affected by the levels of plant available phosphorus in soils. Mean values and 95% confidence intervals of the back-transformed response ratios are shown. Yield response of AMF is highest between 15 and 25 kg and with P solubilizers it is between 25 and 35 kg plant available P per hectare. Yield response in N fixers has its optimum within 45–100 kg and in combination with P solubilizers between 35 and 45 kg plant available P per hectare.

**Figure 6:**
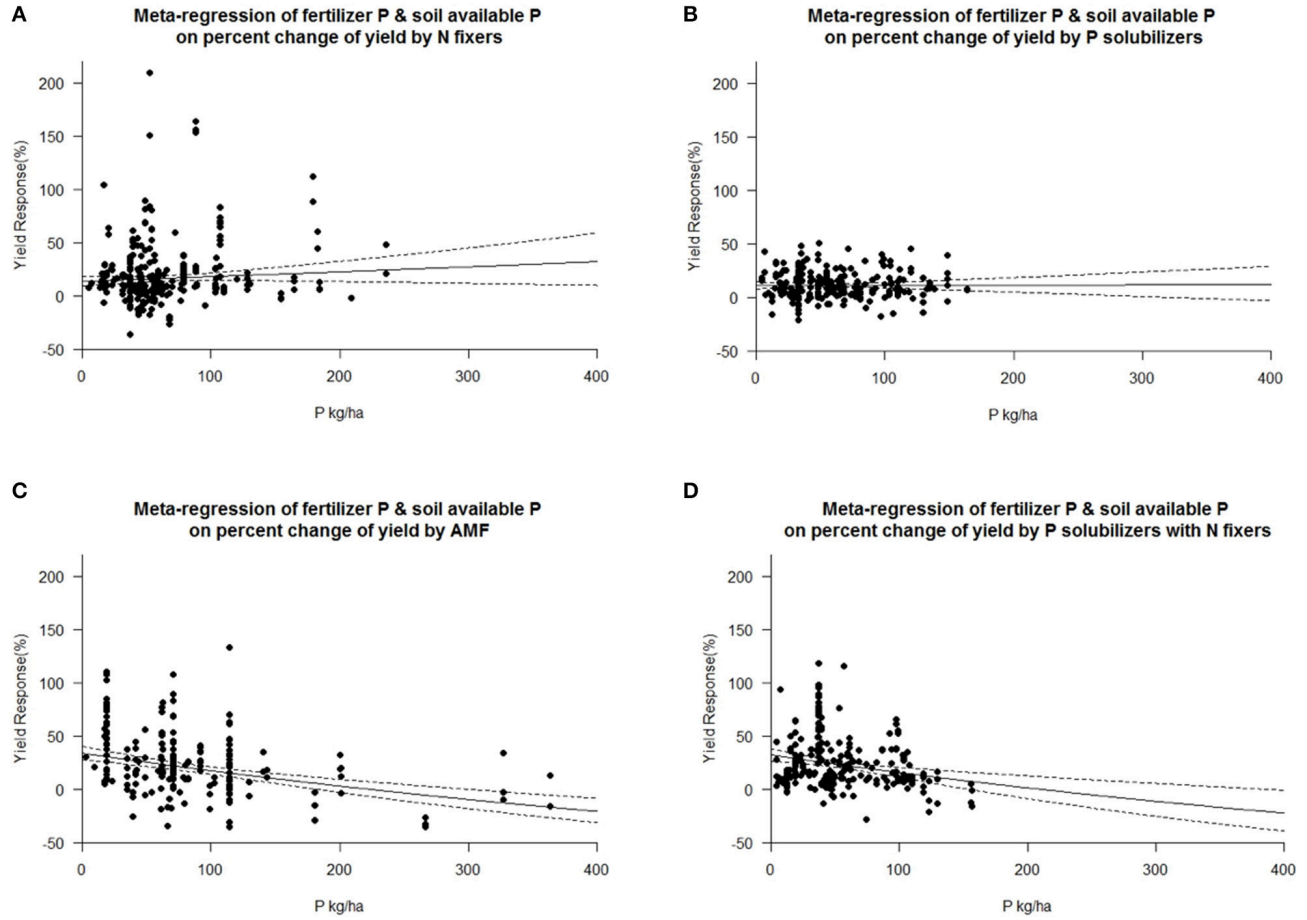
Mixed effects model with fertilizer P added to soil available P as moderator for various biofertilizer categories. Dotted lines depict the confidence interval. **(A)**, *n* = 316, *R*^2^ = 0.08%, *p* = 0.1783 **(B)**, *n* = 255, *R*^2^ = 0%, *p* = 0.9438; **(C)**, *n* = 195, *R*^2^ = 18.74%, *p* = <0.0001; **(D)**, *n* = 230, *R*^2^ = 5.47%, *p* = 0.0002.

Each crop plant, but even crop variety as well as microorganisms have an optimum level of abiotic factors for their physiology and growth. We tested the dependency of biofertilizers with regard to their induced effect size yield under different levels of plant available P, as P is a limiting element for plant growth in many regions of the world. Seven cohorts were formed with the level of plant available phosphorus in soil, which provided sufficient data for comparisons in each level and biofertilizer category. Our results indicate that AMFs have their optimum in yield increase at a low level of 15–25 kg P ha^−1^. P solubilizing microorganisms have their best effect between 25 and 35 kg ha^−1^ soil available P (Figure 5). N fixers alone have an optimum in yield at more than 45 kg ha^−1^ available P; in combination with P solubilizers, this drops to 35–45 kg P ha^−1^ (Figure 5). In their optimum all biofertilizers except P solubilizers increase yield by more than 40%. In a meta-regression with the sum of soil available P and fertilizer P as an explanatory variable, the same increased efficiency at low P levels for AMF and the combined application of P solubilizers and N fixers was found (Figure 6). However, for P solubilizers and N fixers alone no relationship could be found.

### Impact of other biofertilizers

**Figure 7:**
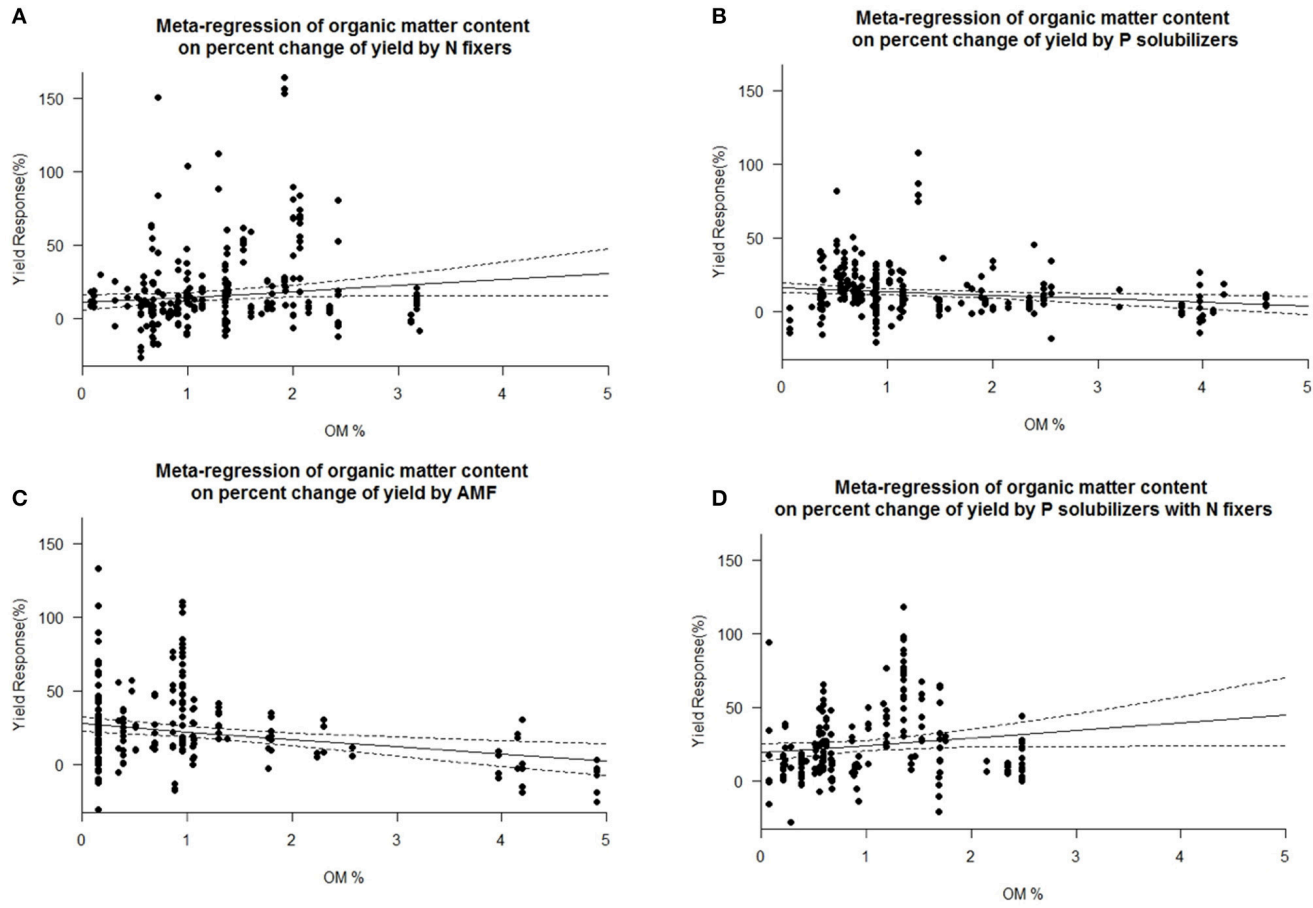
Mixed effects model with organic matter (OM) as moderator for various biofertilizer categories. Dotted lines depict the confidence interval. **(A)**, *n* = 313, *R*^2^ = 0.0%, *p* = 0.9174; **(B)**, *n* = 251, *R*^2^ = 1.96%, *p* = 0.0063; **(C)**, *n* = 202, *R*^2^ = 4.8%, *p* = 0.0007; **(D)**, *n* = 207, *R*^2^ = 2.04%, *p* = 0.0492.

**Figure 8:**
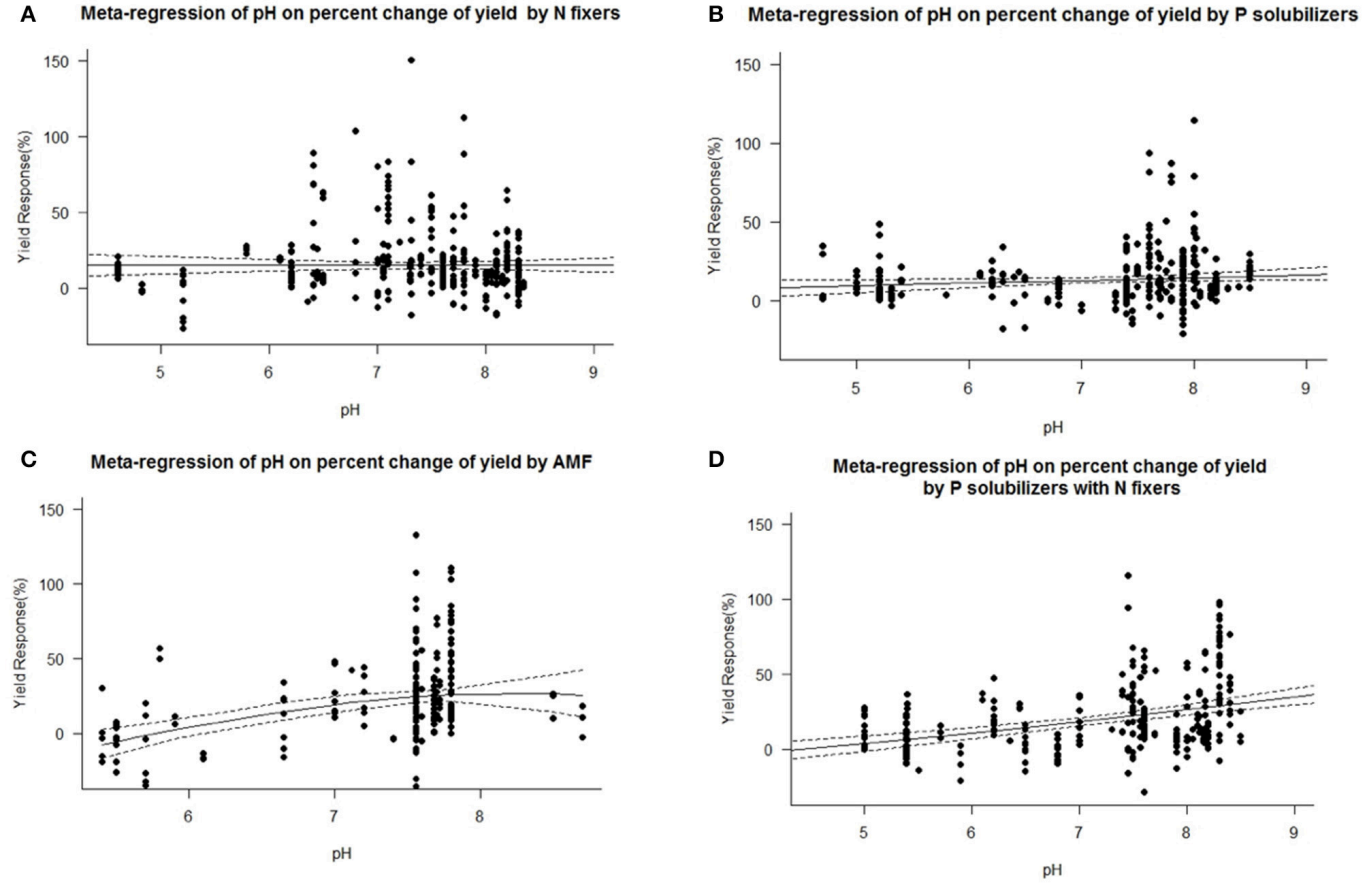
Mixed effects model with pH as moderator for various biofertilizer categories. Dotted lines depict the confidence interval. **(A)**, *n* = 450, *R*^2^ = 0.35%, *p* = 0.2864; **(B)**, *n* = 294, *R*^2^ = 1.19%, *p* = 0.0405; **(C)**, *n* = 206, *R*^2^ = 14.22%, *p* ≤ 0.0001; **(D)**, *n* = 228, *R*^2^ = 13.57%, *p* ≤ 0.0001.

We found a decrease in yield response for P solubilizers and even more for AMF with increased soil organic matter (Figure 7). We also identified pH as an important factor for the success of inoculation of AMF and as well for combined P solubilizers with N fixers (Figure 8D). With AMF there is a slight decrease in yield response at higher pH (Figure 8C).

### Limitations

Meta-analyses face the problem of publication bias. Asymmetry in funnel plots can give information about a publication bias, but its interpretation is sometimes reported to be subjective (Terrin et al., 2005). Our statistical analyses of publication bias resulted in biases to both overly positive and overly negative results, but the bias identified is only moderate, and we thus refrained from adjusting the data to explicitly account for that but we refrain from further interpretation. Regarding variables of potential relevance that have not been covered, the initial soil microbial community had most probably an effect on the inoculation success. Some studies have reported initial populations of their inoculants in the soil, but information on this was too heterogeneous and scarce to be included in this analysis.

## Discussion

### Are biofertilizers a viable option for dryland agriculture?

Our results give strong indications that microbial inoculation is more successful in dry regions. The differences between dry and other climatic conditions are not necessarily thought to be based on microbes conferring drought resistance, but on differences of microbial community in the dry season. Yet microbes are also affected by soil fertility, which is usually lower in dry regions (Thomas et al., 2004). Especially soil organic matter (see Table 4) and soil nitrogen content are reduced. Accordingly, also organic P is lower in drier regions. Phosphorus is highly immobile in soil, particularly in dry soils with less water and less diffusion (Syers et al., 2008). This explains the stronger effect of biofertilizers and especially of P solubilizing bacteria and AMF under these conditions.

When dry soil is suddenly getting wet, there is a burst of availability of N and C, caused by lysis of microorganisms due to the rapid change in water availability (Kieft et al., 1987) and also by the release from non-microbial soil organic carbon (Appel, 1998). More N than C is mineralized which enables microbial degradation of materials with a low C:N ratio and results in further mineralization. This explains the commonly observed pulse of mineralization following wetting of dry and semidry soils (Bloem et al., 1992; Zaady et al., 1996; Cui and Caldwell, 1997; Austin et al., 2004). Both events explain the increased yield effect of biofertilizers under dry climate: Biofertilizers immobilize N to make it available later or directly improve the uptake by plants by facilitating the conversion of ammonium to nitrate and are able to prevent gaseous losses of nitrogen. Other released nutrients may as well be taken up by microbial inoculants and then become plant available later in the season.

Secondly, dry regions are, even with irrigation, still dryer compared to humid areas and often also hotter, causing more evapotranspiration from plants and soil. Biofertilizers like *Azospirillum* may release phytohormones like auxin which enhance root branching and also root elongation. This would be a clear advantage for plants in dry areas (Dobbelaere et al., 1999; Steenhoudt and Vandereyden, 2000). Furthermore, biofertilizers are able to produce other plant hormones like gibberellins and cytokinins in the case of *Azotobacter* (Bhardwaj et al., 2014) reducing stress in the plants and stabilizing their yields. Some bacteria produce ACC deaminase and some biofertilizers are specifically selected for their ability to do so. In stress situations, like drought, plants produce ethylene, which reduces plant growth and may also limit nodulation in leguminous plants. ACC deaminase producing bacteria are able to degrade ethylene thus allowing the plants to grow better by reducing the impact of signal molecules (Shaharoona et al., 2007). Also proline, which accumulates as a common physiological response to various stresses, is degraded by bacteria and improves drought resistance under modest drought (Straub et al., 1997; Verbruggen and Hermans, 2008). This effect was also proven to be agronomically important for plants under drought (Naseem and Bano, 2014; Kumar et al., 2016). Stress situations are more likely in dry regions where also salinity and nutrient deficiencies limit plant growth.

### What are the best biofertilizers?

Our meta-analysis reveals that AMF and combined application of P solubilizers and N fixers are the best inoculants. The higher yield increases by the combinations of the two functional traits N fixation and P solubilization than their separate application suggests an absence of competition and rather synergies between the two traits. Similar numbers for yield increase after inoculation with AMF were found by Lekberg and Koide (2005), who analyzed 290 glasshouse and field trials in a meta-analysis. Berruti et al. (2016) found in their meta-analysis that both yield and plant nutrition were significantly improved by inoculation with AMF under open field conditions in 92% of 112 experiments. In the literature, some microorganisms with the ability to fix nitrogen have been shown to contribute only to a small extent to the N nutrition of crops, and that these results are highly variable (Lee et al., 1994; Bremer et al., 1995; Santi et al., 2013). Our results indicate that their contribution to yield is substantial and with low variation (Figure 3).

Furthermore, a certain amount of plant available P is necessary for all of the biofertilizer groups and none had their optimum at the lowest cohort between 0 and 15 kg ha^−1^ soil available P. In AMF with the best growth promotion at a low level, the growth promotion is well known to depend on the P status of the plant (Smith and Read, 2008a). AMF are able to access phosphorus in soil pores, too small for plant roots, and also extend the access to P in distant soil patches through their hyphal network (Smith and Read, 2008b). Lekberg and Koide (2005) found a greater potential for growth responses in soils with low levels of plant available P in soil, however variability was high. N fixation has large requirements of P and the need is satisfied only at higher levels of P (Graham and Vance, 2000). Leguminous plants for example have developed P solubilizing strategies themselves to satisfy the need of their symbionts. In the meta-analysis by Augusto et al. (2013) it was shown that P availability drives plant growth and also biological nitrogen fixation which explains the strong response at high levels of plant available P in soil in our study. In a meta-regression we have tested furthermore whether our results achieved with soil available P is also found when taking the sum of soil available P and fertilizer P as the explanatory variable. However we found that to result in less of an explanations than before. Considering that only 10–20% of P contained in the crop originates from the most recent fertilization and the remaining 90–80% comes from the reserves accumulated in the soil in earlier fertilizer applications (Sharpley, 1986; McLaughlin et al., 1988), it is no surprise that plant available P in soil is a better control variable.

We are aware of the fact that many biofertilizers may have multiple functions and traits, although not specified by the producers, or by the researchers. Nonetheless we categorized the inoculants to the best of our knowledge. Many studies have used combinations of different biofertilizers and synergistic effects cannot be excluded. Some biofertilizers can fix nitrogen while also solubilizing phosphorus, but they were selected for other traits as well e.g., plant hormone production, solubilization of other nutrients such as Zn or Fe or plant defense [antibiotic 2,4-diacetylphloroglucinol (DAPG), hydrogen cyanide (HCN)]. However, in a separate analysis we found no general superiority to mono inoculation (multi inoculation 15.5 ± 1.4% vs. mono inoculation 16.9 ± 1.3% yield increase). P solubilizers and AMF are most successful at the low levels of plant available P prevalent in soils of tropical regions. Biofertilizers were best in both dry and humid tropics. We also found a decrease in yield response for P solubilizers and even more for AMF with increased soil organic matter (Figure 7), which is likely caused by an increased microbial activity, making it difficult for new microorganisms to establish (Schnürer et al., 1985; Paul, 2016). Also soil organic matter contains organic phosphorus in microbial biomass and other organic pools. We also identified pH as an important factor for the success of inoculation of AMF and as well for combined P solubilizers with N fixers (Figure 8D). Under low and high pH macronutrients are less available for plants. Our results indicate that AMF make only accessable macronutrients at neutral pH more available. Combined P solubilizers and N fixers are effective at high pH. However P solubilizers and N fixers applied alone are independent of pH. With AMF we even found a slight decrease in yield response at higher pH (Figure 8C), which again corresponds to less soluble macronutrients and especially nitrogen and phosphorus.

There is circumstantial evidence why legumes were most responsive to biofertilizers across all effect sizes. Biofertilizers applied to legumes consisted in 12% of all included studies of rhizobia, which were selected to build compatible symbioses with their host plants, but rhizobial inoculum is already present in many soils anyways. Legumes have evolved specific symbioses with N fixing rhizobia but require also other nutrients; reportedly the phosphorus requirement of nodules is up to three times higher than the needs of the surrounding roots (Vadez et al., 1997). Other microorganisms or biofertilizers may help to fulfill this additional nutrient need. In fact, legumes were shown to benefit by an additional AMF inoculation (Mortimer et al., 2008; Omirou et al., 2016). The applied biofertilizers, often with multiple traits such as N fixation and P solubilization, seem to act more synergistically in legumes than in other plants. Interestingly the addition of extra microbial inoculants to sole rhizobia treatments alone improved crop yield also in the range of 19.2% (mean of 59 comparisons from 12 studies), substantiating the synergistic effect between N fixers and P solubilizers.

## Conclusions

We have analyzed three different effect sizes each giving a different perspective on the success of biofertilizers. It was found that dryland agriculture can benefit most from biofertilizers. Due to climate change, in the future there will be even more dryland areas globally. Biofertilizers are thus a promising option for sustainable agriculture. In the future, pretests of the soil community may predict the competitive chance of biofertilizer in a specific soil and help to efficiently produce adapted biofertilizers for each specific application.

## Author contributions

LS, AG, PM, TB, MM, AM, and NM: Designed research; LS: Performed research and analyzed data; LS, PM, AM, TB, and NM: Wrote the paper.

### Conflict of interest statement

The authors declare that the research was conducted in the absence of any commercial or financial relationships that could be construed as a potential conflict of interest.

## Supplementary material

Figure S1 Prisma flow diagram of the literature search.

Figure S2 Funnel plots of the change in yield of **(A)** tropical climate **(B)** dry climate **(C)** continental climate **(D)** oceanic climate. Mean difference of yield on the horizontal axis is plotted against their corresponding standard errors (SE) on the vertical axis.

Supplementary Data Sheet S1 Data used for meta-analysis.

Supplementary Data Sheet S2 Study list used for meta-analysis.
