# Supplementary figures and images for: Improving Crop Yield and Nutrient Use Efficiency via Biofertilization—A Global Meta-analysis
Source: Front Plant Sci. 2018 Jan 12;8:2204. doi: 10.3389/fpls.2017.02204 (PMC5770357; doi:10.3389/fpls.2017.02204)

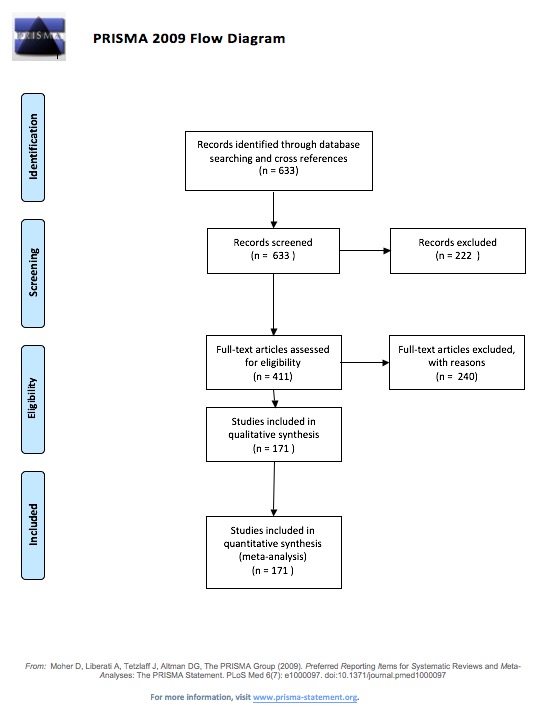

Supplement: Figure S1 — Prisma flow diagram of the literature search. [file Image1.jpeg]

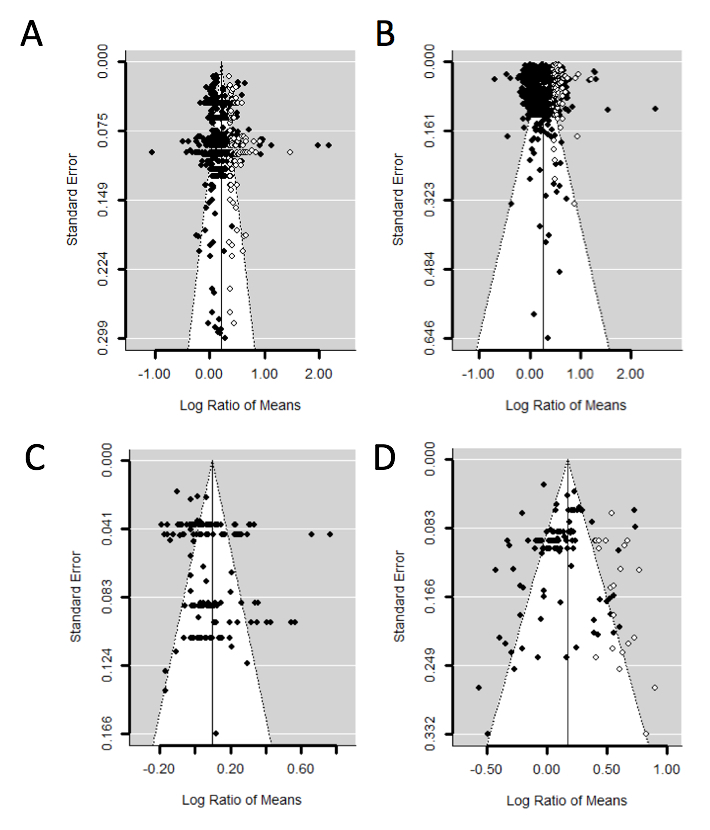

Supplement: Figure S2 — Funnel plots of the change in yield of (A) tropical climate (B) dry climate (C) continental climate (D) oceanic climate. Mean difference of yield on the horizontal axis is plotted against their corresponding standard errors (SE) on the vertical axis. [file Image2.jpeg]
