# Supplementary material for: Improving Crop Yield and Nutrient Use Efficiency via Biofertilization—A Global Meta-analysis
Source: Front Plant Sci. 2018 Jan 12;8:2204. doi: 10.3389/fpls.2017.02204 (PMC5770357; doi:10.3389/fpls.2017.02204)
Supplement: Supplementary Data Sheet S1 — Data used for meta-analysis. [file DataSheet1.docx]

1. Singh AL, Singh PK, Singh PL (1988) Comparative studies on the use of green manuring, organic manuring and Azolla and blue-green-algal biofertilizers to rice. *J Agric Sci* 110:337–343.

2. Mäder P, et al. (2011) Inoculation of root microorganisms for sustainable wheat-rice and wheat-black gram rotations in India. *Soil Biol Biochem* 43(3):609–619.

3. Viruel E, et al. (2014) Inoculation of maize with phosphate solubilizing bacteria: effect on plant growth and yield. *J Soil Sci Plant Nutr* 14(4):819–831.

4. Silva AJN da, et al. (2015) Soil chemical properties and growth of sunflower (Helianthus annuus L.) as affected by the application of organic fertilizers and inoculation with arbuscular mycorrhizal fungi. *Rev Bras Ciência do Solo* 39(1):151–161.

5. Kizilkaya R (2008) Yield response and nitrogen concentrations of spring wheat (Triticum aestivum) inoculated with Azotobacter chroococcum strains. *Ecol Eng* 33(2):150–156.

6. Ahmad W, Shah Z, Khan F, Ali S, Malik W (2013) Maize yield and soil properties as influenced by integrated use of organic, inorganic and bio-fertilizers in a low fertility soil. *Soil Environ* 32(2):121–129.

7. Naseri R, Maleki A, Naserirad H, Shebibi S, Omidian A (2013) Effect of plant growth promoting rhizobacteria (PGPR) on reduction nitrogen fertilizer application in rapeseed (Brassica napus L.). *Middle East J Sci Res* 14(2):213–220.

8. Soleimanzadeh H, Gooshchi F (2013) Effects of azotobacter and nitrogen chemical fertilizer on yield and yield components of wheat (Triticum aestivum L.). *World Appl Sci J* 21(8):1176–1180.

9. Morteza AS, Javad AS (2013) Effect of Nitroxin biofertilizer and nitrogen chemical fertilizer on yield and yield components of rapeseed (Brassica napus L.). *Int J Agric Crop Sci* 6(18):1284–1291.

10. Maheshwari DK, et al. (2012) Integrated approach for disease management and growth enhancement of Sesamum indicum L. utilizing Azotobacter chroococcum TRA2 and chemical fertilizer. *World J Microbiol Biotechnol* 28(10):3015–3024.

11. Salimpour S, Khavazi K, Nadian H, Besharati H, Miransari M (2012) Canola oil production and nutrient uptake as affected by phosphate solubilizing and Sulfur oxidizing bacteria. *J Plant Nutr* 35(13):1997–2008.

12. Qureshi MA, et al. (2012) Role of phosphate solubilizing bacteria (PSB) in enhancing P availability and promoting cotton growth. *J Anim Plant Sci* 22(1):204–210.

13. Jalilian J, Modarres-Sanavy SAM, Saberali SF, Sadat-Asilan K (2012) Effects of the combination of beneficial microbes and nitrogen on sunflower seed yields and seed quality traits under different irrigation regimes. *F Crop Res* 127:26–34.

14. Verma R, Maurya BR, Singh Meena V (2014) Integrated effect of bio-organics with chemical fertilizer on growth, yield and quality of cabbage (Brassica oleracea var capitata). *Dep Soil Sci Agric Chem Indian J Agric Sci* 84(8):914–9.

15. Yazdani M, Bagheri H, Ghanbari-Malidarreh A (2011) Evaluation effects of P-solubilizer (PSM) and plant growth promoting rhizobacteria (PGPR) on morphologic indices of corn (Zea mays L.). *Adv Environ Biol* 5(13):3782–3786.

16. Yolcu H, Turan M, Lithourgidis A, Çakmakçi R, Koç A (2011) Effects of plant growth-promoting rhizobacteria and manure on yield and quality characteristics of Italian ryegrass under semi arid conditions. *Aust J Crop Sci* 5(13):1730–1736.

17. Sharifi RS, Khavazi K (2011) Effect of seed priming with plant growth promoting rhizobacteria (PGPR) on dry matter accumulation and yield of maize (Zea mays L.) hybrids. *J Food, Agric Environ* 9(3&4):496–500.

18. Akbari P, Ghalavand A, Modares Sanavy AM, Aghaalikhani M, Shoghi Kalkhoran S (2011) Comparison of different nutritional levels and the effect of plant growth promoting rhizobacteria (PGPR) on the grain yield and quality of sunflower. *Aust J Crop Sci* 5(12):1570–1576.

19. El-Yazeid AA (2011) Enhancing growth, productivity and quality of tomato plants using phosphate solubilizing microorganisms. *Aust J Basic Appl Sci 5(7) 371-379, 2011 ISSN 1991-8178* 5(7):371–379.

20. Rokhzadi A, Toashih V (2011) Nutrient uptake and yield of chickpea (Cicer arietinum L .) inoculated with plant growth- promoting rhizobacteria. *J Plant Nutr* 5(1):44–48.

21. Ekin Z (2010) Performance of phosphate solubilizing bacteria for improving growth and yield of sunflower (Helianthus annuus L .) in the presence of phosphorus fertilizer. *African J Biotechnol* 9(25):3794–3800.

22. Prvulović D, Popović M, Malenčić D, Marinković B, Jaćimović G (2009) Effects of nitrogen fertilization on the biochemical and physiological parameters in leaves and root of sugar beet associated with Azotobacter chroococcum. *J Plant Nutr* 33(1):15–26.

23. Suman A, Singh P, Lal M (2013) Effects of diverse habitat biofertilizers on yield and nitrogen balance in plant-ratoon crop cycle of sugarcane in subtropics. *Sugar Tech* 15(1):36–43.

24. Gamal-Eldin H, Elbanna K (2011) Field evidence for the potential of Rhodobacter capsulatus as biofertilizer for flooded rice. *Curr Microbiol* 62(2):391–395.

25. Valverde A, et al. (2006) Differential effects of coinoculations with Pseudomonas jessenii PS06 (a phosphate-solubilizing bacterium) and Mesorhizobium ciceri C-2/2 strains on the growth and seed yield of chickpea under greenhouse and field conditions. *Plant Soil* 287(1–2):43–50.

26. Dutta D, Bandyopadhyay P (2009) Performance of chickpea (Cicer arietinum L.) to application of phosphorus and bio-fertilizer in laterite soil. *Arch Agron Soil Sci* 55(2):147–155.

27. Azzaz NA, Hassan EA, Hamad EH (2009) The chemical constituent and vegetative and yielding characteristics of fennel plants treated with organic and bio-fertilizer instead of mineral fertilizer. *Aust J Basic Appl Sci* 3(2):579–587.

28. Ahmad R, Arshad M, Khalid A, Zahir AZ (2008) Organic farming in Spain - Two case studies. *J Sustain Agric* 31(4):57–77.

29. Singh G, Sekhon HS, Sharma P (2011) Effect of irrigation and biofertilizer on water use, nodulation, growth and yield of chickpea (Cicer arietinum L .). *Arch Agron Soil Sci* 57(7):715–726.

30. Cong PT, et al. (2011) Effects of a multistrain biofertilizer and phosphorus rates on nutrition and grain yield of paddy rice on a sandy soil in southern Vietnam. *J Plant Nutr* 34(7):1058–1069.

31. Salinas-Ramirez N, Escalante-Estrada JA, Rodriguez-Gonzalez MT, Sosa-Montez E (2011) Yield and nutritional quality of snap bean in terms of biofertilization. *Trop Subtrop Agroecosystems* 13:347–355.

32. Namvar A, Khandan T (2013) Response of wheat to mineral nitrogen fertilizer and biofertilizer (Azotobacter sp. and Azospirillum sp.) inoculation under different levels of weed interference. *Ekologija* 59(2):85–94.

33. Darzi M, Hadi MHS (2012) Effects of the application of organic manure and biofertilizer on the fruit yield and yield components in Dill (Anethum graveolens). *J Med Plants Res* 6(16):3266–3271.

34. Naserirad H, Soleymanifard A, Naseri R (2011) Effect of integrated application of bio-fertilizer on grain yield, yield components and associated traits of maize cultivars. *Am Eurasian JAgric& EnvironSci* 10(2):271–277.

35. Haque M a, Khan MK (2012) Effects of phosphatic biofertilizer with inorganic and organic sources of phosphorus on growth and yield of lentil. *J Environ Sci Nat Resour* 5(2):225–230.

36. Wijebandara DMDI, Dasog GS, Patil PL, Hebbar M (2008) Effect of nutrient levels on rice (Oryza sativa L.) under System of Rice Intensification (SRI) and traditional methods of cultivation. *Trop Agric Res* 20(1):343–353.

37. Mobasser HR, Moradgholi A (2012) Mycorrhizal bio-fertilizer applications on yield seed corn varieties in Iran. *Ann Biol Res* 3(2):1109–1116.

38. Kecskés ML, et al. (2015) Effects of bacterial inoculant biofertilizers on growth, yield and nutrition of rice in Australia. *J Plant Nutr* 4167(October):00–00.

39. Kumar M, Bauddh K, Sainger M, Sainger PA, Singh RP (2015) Enhancing efficacy of Azotobactor and Bacillus by entrapping in organic matrix for rice cultivation. *Agroecol Sustain Food Syst* 39(8):907–923.

40. Ozturk A, Caglar O, Sahin F (2003) Yield response of wheat and barley to inoculation of plant growth promoting rhizobacteria at various levels of nitrogen fertilization. *J Plant Nutr Soil Sci* 166(2):262–266.

41. El-Sirafy ZM, Woodard HJ, El-Norjar EM (2006) Contribution of biofertilizers and fertilizer nitrogen to nutrient uptake and yield of Egyptian winter wheat. *J Plant Nutr* 29(4):587–599.

42. Moslemi Z, et al. (2012) Effects of super absorbent polymer and plant growth promoting rhizobacteria on yield and yield components of maize under drought stress and normal conditions. *Am J Agric Environ Sci* 12(3):358–364.

43. Feleafel MN, Mirdad ZM (2014) Influence of organic nitrogen on the snap bean grown in sandy soil. *Int J Agric Biol* 16(1):65–72.

44. Chatterjee R, Jana JC, Paul PK (2012) Enhancement of head yield and quality of cabbage (Brassica oleracea) by combining different sources of nutrients. *Indian J Agric Sci* 82(4):323–327.

45. Gan Y, Hanson KG, Zentner RP, Selles F, McDonald CL (2005) Response of lentil to microbial inoculation and low rates of fertilization in the semiarid Canadian prairies. *Can J Plant Sci* 85:847–855.

46. Nguyen HT, Deaker R, Kennedy IR, Roughley RJ (2003) The positive yield response of field-grown rice to inoculation with a multi-strain biofertiliser in the Hanoi area, Vietnam. *Symbiosis* 35(1–3):231–245.

47. Mondal T, Datta JK, Mondal NK (2014) An alternative eco-friendly approach for sustainable crop production with the use of indigenous inputs under old alluvial soil zone of Burdwan, West Bengal, India. *Arch Agron Soil Sci* 340(July 2014):1–18.

48. Dalal LP, Nandkar PB (2010) Effect of biofertilisers and NPK on Abelmoschus esculentus (L .) in relation to fruit yield. *The Bioscan* 5(2):309–311.

49. Al-Karaki G, McMichael B, Zak J (2004) Field response of wheat to arbuscular mycorrhizal fungi and drought stress. *Mycorrhiza* 14(4):263–269.

50. Shaharoona B, Naveed M, Arshad M, Zahir ZA (2008) Fertilizer-dependent efficiency of Pseudomonads for improving growth, yield, and nutrient use efficiency of wheat (Triticum aestivum L.). *Appl Microbiol Biotechnol* 79(1):147–155.

51. Elkoca E, Kantar F, Sahin F (2007) Influence of nitrogen fixing and phosphorus solubilizing bacteria on the nodulation, plant growth, and yield of chickpea. *J Plant Nutr* 31:157–171.

52. Van VT, Berge O, Kê SN, Balandreau J, Heulin T (2000) Repeated beneficial effects of rice inoculation with a strain of Burkholderia vietnamiensis on early and late yield components in low fertility sulphate acid soils of Vietnam. *Plant Soil* 218:273–284.

53. Naveed M, Khalid M, Jones DL, Ahmad R, Zahir ZA (2008) Relative efficacy of Pseudomonas spp., containing Acc-deaminase for improving growth and yield of maize (Zea mays L.) in the presence of organic fertilizer. *Pakistan J Bot* 40:1243–1251.

54. Shaharoona B, Jamro GM, Zahir ZA, Arshad M, Memon KS (2007) Effectiveness of various Pseudomonas spp. and Burkholderia caryophylli containing ACC-deaminase for improving growth and yield of wheat (Triticum aestivum L.). *J Microbiol Biotechnol* 17(8):1300–1307.

55. Iqbal MA, et al. (2012) Integrated use of Rhizobium leguminosarum, plant growth promoting rhizobacteria and enriched compost for improving growth, nodulation and yield of lentil (Lens culinaris Medik.). *Chil J Agric Res* 72(March):104–110.

56. Jilani G, et al. (2007) Enhancing crop growth, nutrients availability, economics and beneficial rhizosphere microflora through organic and biofertilizers. *Ann Microbiol* 57(2):177–184.

57. Cozzolino V, Di Meo V, Piccolo A (2013) Impact of arbuscular mycorrhizal fungi applications on maize production and soil phosphorus availability. *J Geochemical Explor* 129:40–44.

58. El Zemrany H, et al. (2006) Field survival of the phytostimulator Azospirillum lipoferum CRT1 and functional impact on maize crop, biodegradation of crop residues, and soil faunal indicators in a context of decreasing nitrogen fertilisation. *Soil Biol Biochem* 38(7):1712–1726.

59. Pedraza RO, Bellone CH, Carrizo de Bellone S, Boa Sorte PMF, Teixeira KR dos S (2009) Azospirillum inoculation and nitrogen fertilization effect on grain yield and on the diversity of endophytic bacteria in the phyllosphere of rice rainfed crop. *Eur J Soil Biol* 45(1):36–43.

60. Naiman AD, Latrónico A, García de Salamone IE (2009) Inoculation of wheat with Azospirillum brasilense and Pseudomonas fluorescens: Impact on the production and culturable rhizosphere microflora. *Eur J Soil Biol* 45(1):44–51.

61. Sharma SN, Prasad R (2003) Yield and P uptake by rice and wheat grown in a sequence as influenced by phosphate fertilization with diammonium phosphate and Mussoorie rock phosphate with or without crop residues and phosphate solubilizing bacteria. *J Agric Sci* 141:359–369.

62. Saini VK, Bhandari SC, Tarafdar JC (2004) Comparison of crop yield, soil microbial C, N and P, N-fixation, nodulation and mycorrhizal infection in inoculated and non-inoculated sorghum and chickpea crops. *F Crop Res* 89(1):39–47.

63. Singh H, Reddy SM (2012) Improvement of wheat and maize crops by inoculating Aspergillus spp. in alkaline soil fertilized with rock phosphate. *Arch Agron Soil Sci* 58(5):535–546.

64. Kannaiyan S, Aruna SJ, Merina Prem Kumari S, Hall DO (1997) Immobilized cyanobacteria as a biofertilizer for rice crops. *J Appl Phycol* 9(2):167–174.

65. Prasanna R, et al. (2013) Evaluating the establishment and agronomic proficiency of cyanobacterial consortia as organic options in wheat–rice cropping sequence. *Exp Agric* 49(3):416–434.

66. Rana A, Joshi M, Prasanna R, Shivay YS, Nain L (2012) Biofortification of wheat through inoculation of plant growth promoting rhizobacteria and cyanobacteria. *Eur J Soil Biol* 50:118–126.

67. Stefan M, Munteanu N, Stoleru V, Mihasan M, Hritcu L (2013) Seed inoculation with plant growth promoting rhizobacteria enhances photosynthesis and yield of runner bean (Phaseolus coccineus L.). *Sci Hortic (Amsterdam)* 151:22–29.

68. Joe MM, et al. (2012) Survival of Azospirillum brasilense flocculated cells in alginate and its inoculation effect on growth and yield of maize under water deficit conditions. *Eur J Soil Biol* 50:198–206.

69. Prasanna R, et al. (2015) Prospecting cyanobacteria-fortified composts as plant growth promoting and biocontrol agents in cotton. *Exp Agric* 51(1):42–65.

70. Prasanna R, et al. (2015) Cyanobacterial inoculation in rice grown under flooded and SRI modes of cultivation elicits differential effects on plant growth and nutrient dynamics. *Ecol Eng* 84:532–541.

71. Ghosh TK, Saha KC (1997) Effects of inoculation of cyanobacteria on nitrogen status and nutrition of rice (Oryza sativa L.) in an Entisol amended with chemical and organic sources of nitrogen. *Biol Fertil Soils* 24(1):123–128.

72. Rosas SB, et al. (2009) Root colonization and growth promotion of wheat and maize by Pseudomonas aurantiaca SR1. *Soil Biol Biochem* 41(9):1802–1806.

73. Díaz-Zorita M, Fernández-Canigia MV (2009) Field performance of a liquid formulation of Azospirillum brasilense on dryland wheat productivity. *Eur J Soil Biol* 45(1):3–11.

74. Verma JP, Yadav J, Tiwari KN, Kumar A (2013) Effect of indigenous Mesorhizobium spp. and plant growth promoting rhizobacteria on yields and nutrients uptake of chickpea (Cicer arietinum L.) under sustainable agriculture. *Ecol Eng* 51:282–286.

75. Colla G, Rouphael Y, Bonini P, Cardelli M (2015) Coating seeds with endophytic fungi enhances growth, nutrient uptake, yield and grain quality of winter wheat. *Int J Plant Prod* 9(2):171–190.

76. Hameeda B, Harini G, Rupela OP, Wani SP, Reddy G (2008) Growth promotion of maize by phosphate-solubilizing bacteria isolated from composts and macrofauna. *Microbiol Res* 163(2):234–242.

77. Yadegari M, Rahmani HA (2010) Evaluation of bean (Phaseolus vulgaris) seeds inoculation with Rhizobium phaseoli and plant growth promoting rhizobacteria on yield and yield components. *African J Agric Res* 5(9):792–799.

78. Hungria M, Campo RJ, Souza EM, Pedrosa FO (2010) Inoculation with selected strains of Azospirillum brasilense and A. lipoferum improves yields of maize and wheat in Brazil. *Plant Soil* 331(1):413–425.

79. Puente ML, et al. (2013) Plant-growth promotion of Argentinean isolates of Azospirillum brasilense on rice (Oryza sativa l .) under controlled and field conditions. *Am J Agric Environ Sci* 13(10):1361–1369.

80. Araújo É de O, Mercante FM, Vitorino ACT (2015) Effect of nitrogen fertilization associated with inoculation of Azospirillum brasilense and Herbaspirillum seropedicae on corn. *African J Agric Res* 10(3):137–145.

81. Meena RK, Singh YV, Bana RS, Lata (2013) Effect of nitrogen, compost and plant growth promoting rhizobactria on yield and nutrient uptake by rice (Oryza sativa). *Indian J Agron* 58(3):424–426.

82. Kumar A, Maurya BR, Raghuwanshi R (2015) Characterization of bacterial strains and their impact on plant growth promotion and yield of wheat and microbial populations of soil. *African J Agric Res* 10(12):1367–1375.

83. Reynders L, Vlassak K (1982) Use of Azospirillum brasilense as biofertilizer in intensive wheat cropping. *Plant Soil* 66(2):217–223.

84. Kapulnik Y, Okon Y, Henis Y (1987) Yield response of spring wheat cultivars (Triticum aestivum and T. turgidum) to inoculation with Azospirillum brasilense under field conditions. *Biol Fertil Soils* 4(1–2):27–35.

85. Davaran-Hagh E, Mirshekari B, Reza-Ardakani M, Farahvash F, Rejali F (2015) Azospirillum lipoferum and nitrogen fertilization effect on chlorophyll content, nutrients uptake and biometric properties of Zea mays L. *Agrociencia* 49(8):889–897.

86. Goos RJ, Johnson BE, Stack RW (1994) Penicillium bilaji and phosphorus fertilization effects on the growth, development, yield and common root rot severity of spring wheat. *Fertil Res* 39(2):97–103.

87. Nandakumar R, et al. (2001) A new bio-formulation containing plant growth promoting rhizobacterial mixture for the management of sheath blight and enhanced grain yield in rice. *BioControl* 46(4):493–510.

88. Kumar R, Chandra R (2008) Influence of PGPR and PSB on Rhizobium leguminosarum Bv . viciae strain competition and symbiotic performance in lentil. *Crop Res* 4(3):297–301.

89. Paul S, Rathi MS, Tyagi SP (2011) Interactive effect with AM fungi and Azotobacter inoculated seed on germination, plant growth and yield in cotton (Gossypium hirsutum). *Indian J Agric Sci* 81(11):1041–1045.

90. Schultz N, et al. (2014) Inoculation of sugarcane with diazotrophic bacteria. *Rev Bras Ciência do Solo* 38(2):407–414.

91. Suri VK, Choudhary AK (2013) Effects of vesicular arbuscular mycorrhizae and applied phosphorus through targeted yield precision model on root morphology, productivity, and nutrient dynamics in soybean in an acid Alfisol. *Commun Soil Sci Plant Anal* 44(17):2587–2604.

92. Anandham R, et al. (2007) Potential for plant growth promotion in groundnut (Arachis hypogaea L.) cv. ALR-2 by co-inoculation of sulfur-oxidizing bacteria and Rhizobium. *Microbiol Res* 162(2):139–153.

93. Bernabeu PR, et al. (2015) Colonization and plant growth-promotion of tomato by Burkholderia tropica. *Sci Hortic (Amsterdam)* 191:113–120.

94. Luna MF, Aprea J, Crespo JM, Boiardi JL (2012) Colonization and yield promotion of tomato by Gluconacetobacter diazotrophicus. *Appl Soil Ecol* 61:225–229.

95. Gholami A, Nezarat S (2008) The effect of plant growth promoting rhizobacteria (PGPR) on germination, seedling growth and yield of maize. *Pakistan J Biol Sci* 37:1–7.

96. Gravel V, Antoun H, Tweddell RJ (2007) Growth stimulation and fruit yield improvement of greenhouse tomato plants by inoculation with Pseudomonas putida or Trichoderma atroviride: Possible role of indole acetic acid (IAA). *Soil Biol Biochem* 39(8):1968–1977.

97. Anitha KG, Thangaraju M (2010) Influence of N fertilization on colonization and activity of Gluconacetobacter diazotrophicus in Sugarcane. *J AgroCrop Sci* 1(1):6–11.

98. Nzanza B, Marais D, Soundy P (2011) Response of tomato (Solanum lycopersicum L.) to nursery inoculation with Trichoderma harzianum and arbuscular mycorrhizal fungi under field conditions. *Acta Agric Scand Sect B - Soil Plant Sci* 62(3):209–215.

99. Douds Jr. DD, Reider C (2003) Inoculation with mycorrhizal fungi increases the yield of green peppers in a high P soil. *Biol Agric Hortic* 21:91–102.

100. Ortas I (2012) The effect of mycorrhizal fungal inoculation on plant yield, nutrient uptake and inoculation effectiveness under long-term field conditions. *F Crop Res* 125:35–48.

101. Conversa G, Lazzizera C, Bonasia A, Elia A (2012) Yield and phosphorus uptake of a processing tomato crop grown at different phosphorus levels in a calcareous soil as affected by mycorrhizal inoculation under field conditions. *Biol Fertil Soils* 49(6):691–703.

102. Dey R, Pal KK, Bhatt DM, Chauhan SM (2004) Growth promotion and yield enhancement of peanut (Arachis hypogaea L.) by application of plant growth-promoting rhizobacteria. *Microbiol Res* 159(4):371–394.

103. Sarathambal C, Ilamurugu K, Balachandar D, Chinnadurai C, Gharde Y (2015) Characterization and crop production efficiency of diazotrophic isolates from the rhizosphere of semi-arid tropical grasses of India. *Appl Soil Ecol* 87:1–10.

104. Baig KS, Arshad M, Khalid A, Hussain S (2014) Improving growth and yield of maize through bioinoculants carrying auxin production and phosphate solubilizing activity. *Soil Environ* 33(2):159–168.

105. Ortas I (2010) Effect of mycorrhiza application on plant growth and nutrient uptake in cucumber production under field conditions. *Spanish J Agric Res* 8:S116–S122.

106. Subramanian KS, Santhanakrishnan P, Balasubramanian P (2006) Responses of field grown tomato plants to arbuscular mycorrhizal fungal colonization under varying intensities of drought stress. *Sci Hortic (Amsterdam)* 107(3):245–253.

107. Gholamhoseini M, Ghalavand A, Dolatabadian A, Jamshidi E, Khodaei-Joghan A (2013) Effects of arbuscular mycorrhizal inoculation on growth, yield, nutrient uptake and irrigation water productivity of sunflowers grown under drought stress. *Agric Water Manag* 117:106–114.

108. Erman M, et al. (2011) Effects of Rhizobium, arbuscular mycorrhiza and whey applications on some properties in chickpea (Cicer arietinum L.) under irrigated and rainfed conditions 1-Yield, yield components, nodulation and AMF colonization. *F Crop Res* 122(1):14–24.

109. Celebi SZ, Demir S, Celebi R, Durak ED, Yilmaz IH (2010) The effect of arbuscular mycorrhizal fungi (AMF) applications on the silage maize (Zea mays L.) yield in different irrigation regimes. *Eur J Soil Biol* 46(5):302–305.

110. Khaliq A, Sanders FE (2000) Effects of vesicular-arbuscular mycorrhizal inoculation on the yield and phosphorus uptake of field-grown barley. *Soil Biol Biochem* 32(11–12):1691–1696.

111. Prakamhang J, et al. (2014) Proposed some interactions at molecular level of PGPR coinoculated with Bradyrhizobium diazoefficiens USDA110 and B. japonicum THA6 on soybean symbiosis and its potential of field application. *Appl Soil Ecol* 85:38–49.

112. Candido V, et al. (2015) Growth and yield promoting effect of artificial mycorrhization on field tomato at different irrigation regimes. *Sci Hortic (Amsterdam)* 187:35–43.

113. Carlier E, Rovera M, Rossi Jaume A, Rosas SB (2008) Improvement of growth, under field conditions, of wheat inoculated with Pseudomonas chlororaphis subsp. aurantiaca SR1. *World J Microbiol Biotechnol* 24(11):2653–2658.

114. Fulchieri M, Frioni L (1994) Azospirillum inoculation on maize (Zea mays): effect on yield in a field experiment in central argentina. *Soil Biol Biochem* 26(7):921–923.

115. Mansotra P, Sharma P, Sharma S (2015) Bioaugmentation of Mesorhizobium cicer, Pseudomonas spp. and Piriformospora indica for sustainable chickpea production. *Physiol Mol Biol Plants* 21(3):385–393.

116. Imran A, Mirza MS, Shah TM, Malik KA, Hafeez FY (2015) Differential response of kabuli and desi chickpea genotypes toward inoculation with PGPR in different soils. *Front Microbiol* 6(AUG):1–14.

117. Raverkar K, Konde B (1988) Effect of Rhizobium and Azospirillum lipoferum inoculation on the nodulation, yield and nitrogen uptake of peanut cultivars. *Plant Soil* 106:249–252.

118. Germida JJ, Walley FL (1996) Plant growth-promoting rhizobacteria alter rooting patterns and arbuscular mycorrhizal fungi colonization of field-grown spring wheat. *Biol Fertil Soils* 23(2):113–120.

119. Hellal F, Mahfouz S (2011) Partial substitution of mineral nitrogen fertilizer by bio-fertilizer on (Anethum graveolens L.) plant. *Agric Biol J North Am* 2(4):652–660.

120. Saha KC, Sannigrahi S, Mandal LN (1985) Effect of inoculation of Azospirillum lipoferum on nitrogen fixation in rhizosphere soil, their association with root, yield and nitrogen uptake by mustard (Brassica juncea). *Plant Soil* 87(2):273–280.

121. de Freitas JR, Germida JJ (1992) Growth promotion of winter wheat by fluorescent pseudomonads under field conditions. *Soil Biol Biochem* 24(11):1137–1146.

122. Millet E, Avivi Y, Feldman M (1985) Effects of rhizospheric bacteria on wheat yield under field conditions. *Plant Soil* 86:347–355.

123. Kapulnik Y, Sarig S, Nur I, Okon Y (1983) Effect of Azospirillum inoculation on yield of field grown wheat. *Can J Microbiol* 29(Okon 1982):895–899.

124. Sharma SN, Ray SB, Pandey SL, Prasad R (1983) Effect of irrigation, pyrites and phosphobacteria on the efficiency of rock phosphate applied to lentils. *J Agric Sci* 101:467–472.

125. Yanni YG (1992) The effect of cyanobacteria and Azolla on the performance of rice under different levels of fertilizer nitrogen. *World J Microbiol Biotechnol* 8(2):132–136.

126. Chattoo MA, et al. (2007) Response of garlic (Allium sativum L.) to biofertilizer application. *Asian J Hortic* 2(2):249–252.

127. El-Kholy MA, El-Ashry S, Gomaa AM (2005) Biofertilization of maize crop and its impact on yield and grains nutrient content under low rates of mineral fertilizers. *J Appl Sci Res* 1(2):117–121.

128. Serfling A, Wirsel SGR, Lind V, Deising HB (2007) Performance of the biocontrol fungus Piriformospora indica on wheat under greenhouse and field conditions. *Phytopathology* 97(4):523–531.

129. Abdel Latef AAH, Chaoxing H (2011) Effect of arbuscular mycorrhizal fungi on growth, mineral nutrition, antioxidant enzymes activity and fruit yield of tomato grown under salinity stress. *Sci Hortic (Amsterdam)* 127(3):228–233.

130. Pal UR, Malik HS (1981) Contribution of Azospirillum brasilense to the nitrogen needs of sorghum (Sorghum bicolor (L.) Moench.) in humid sub-tropics. *Plant Soil* 63:501–504.

131. Rai R (1985) Studies on nitrogen fixation by Machete-resistant mutant strains of Azospirillum brasilense: their associative -N2-fixation and yield response of rice to root inoculation in calcareous soil. *J Gen Appl Microbiol* 31:1–16.

132. Singh RK, Agarwal RL, Singh SK (2007) Integrated nutrient management in wheat (Triticum aestivum). *Ann Agric Res New Ser* 28(1):20–24.

133. García De Salomone I, Döbereiner J (1996) Maize genotype effects on the response to Azospirillum inoculation. *Biol Fertil Soils* 21(3):193–196.

134. Swędrzyńska D, Sawicka A (2000) Effect of inoculation with Azospirillum brasilense on development and yielding of winter wheat and oat under different cultivation conditions. *Polish J Environ Stud* 9(6):505–509.

135. Khorshidi YR, Ardakani MR, Ramezanpour MR, Khavazi K, Zargari K (2011) Response of yield and yield components of rice (Oryza sativa L.) to Pseudomonas flouresence and Azospirillum lipoferum under different nitrogen levels. *Am J Agric Environ Sci* 10(3):387–395.

136. Choudhary GR, Jain NK, Jat NL (2008) Response of coriander (Coriandrum sativum) to inorganic nitrogen, farmyard manure and biofertilizer. *Indian J Agric Sci* 78(9):761–763.

137. Davari M, Sharma SN, Mirzakhani M (2012) Residual influence of organic materials, crop residues, and biofertilizers on performance of succeeding mung bean in an organic rice-based cropping system. *Int J Recycl Org Waste Agric* 1(1):14.

138. Janagard MS, Raei Y, Gasemi-Golezani K, Aliasgarzad N (2013) Soybean response to biological and chemical fertilizers. *Int J Agric Crop Sci* 5(3):261–266.

139. Datta M, Banik S (1994) Effect of poultry manure and phosphate-dissolving bacteria on rice (Oryza sativa) in acid soil. *Indian J Agric Sci* 64(11):791–793.

140. Purbey SK, Sen NL (2005) Effect of bioinoculants and bioregulators on productivity and quality of fenugreek (Trigonella foenum-graecum). *Indian J Agric Sci* 75(9):608–611.

141. Majumdar B, Venkatesh MS, Saha R (2007) Effect of nitrogen, farmyard manure and non-symbiotic nitrogen-fixing bacteria on yield, nutrient uptake and soil fertility in upland rice (Oryza sativa). *Indian J Agric Sci* 77(6):335–339.

142. Senapati HK, Pal AK, Samant PK (2005) Effect of chemical fertilizer, organic manure, lime and biofertilizer on yield of turmeric (Curcuma longa). *Indian J Agric Sci* 75(9):593–595.

143. Sharma SK (2002) Effect of Azospirillum, Azotobacter and nitrogen on growth and yield of cabbage (Brassica oleracea var capitata). *Indian J Agric Sci* 72(9):555–557.

144. Upadhyay AK, Bahadur A, Singh J (2012) Effect of organic manures and biofertilizers on yield, dry matter partitioning and quality traits of cabbage (Brassica oleracea var. capitata). *Indian J Agric Sci* 82(1):31–34.

145. Pathak AK, Godika S (2010) Effect of organic fertilizers, biofertilizers, antagonists and nutritional supplements on yield and disease incidence in Indian mustard in arid soil. *Indian J Agric Sci* 80(7):652–654.

146. Kumpawat BS (2010) Integrated nutrient management in blackgram (Vigna mungo) and its residual effect on succeeding mustard (Brassica juncea) crop. *Indian J Agric Sci* 80(1):76–79.

147. Singh K (1999) Effect of bio-fertilizers and phosphorus levels on the pruduction of potato (Solanum tuberosum) crop under north-east hill conditions. *Indian J Agric Sci* 69(10):746–749.

148. Jain K, Jat NL, Choudhary GR (2007) Response of fennel (Foeniculum vulgare) to inorganic nitrogen, farmyard manure and Azospirillum. *Indian J Agric Sci* 77(6):376–378.

149. Dubey SK, Agarwal S (1999) Effect of phosphate-solubilizing micro-organisms as single and composite inoculant on rainfed soybean (Glycine max) in Vertisol. *Indian J Agric Sci* 69(8):611–613.

150. Kumari C, Mankar A, Karuna K, Solankey SS, Singh VK (2015) Effect of different levels of nitrogen and microbial inoculants on yield and quality of cabbage (Brassica Oleracea Var. Capitata L.) cv Pride of India. *Indian J Agric Sci* 85(4):515–518.

151. Choudhary RL, et al. (2010) Performance of rice (Oryza sativa) hybrids grown by the system of rice intensification with plant growth-promoting rhizobacteria. *Indian J Agric Sci* 80(10):917–920.

152. Khanna V, Sharma P (2011) Potential for enhancing lentil (Lens culinaris) productivity by co-inoculation with PSB, plant growth-promoting rhizobacteria and Rhizobium. *Indian J Agric Sci* 81(10):932–934.

153. Kumar A, Sharma KD, Gera R (2011) Arbuscular mycorrhizae (Glomus mosseae) symbiosis for increasing the yield and quality of wheat (Triticum aestivum). *Indian J Agric Sci* 81(5):478–480.

154. Singh GP, Singh PL, Panwar AS (2011) Response of ground nut (Arachis hypogaea) to biofertilizer, groundnut organic and inorganic sources of nutrient in North East India. *Legum Res* 34(3):196–201.

155. Ghosh DC, Mohiuddin M (2000) Response of summer sesame (Sesamum indicum) to biofertilizer and growth regulator. *Agric Sci Dig* 20(2):90–92.

156. Ghosh DC, Nandi P, Shivkumar K (2000) Effect of biofertilizer and growth regulator on growth and productivity of potato (Solanum tuberosum) at different fertility, levels. *Indian J Agric Sci* 70(7):466–468.

157. Thakur SK, Jha CK, Kumari G, Singh VP (2010) Effect of Trichoderma inoculated trash, nitrogen level and biofertilizer on performance of sugarcane (Saccharum officinarum) in calcareous soils of Bihar. *Indian J Agron* 55(4):308–311.

158. Gawai PP, Pawar VS (2006) Integrated nutrient management in sorghum (Sorghum bicolor) – chickpea (Cicer arietinum) cropping sequence under irrigated conditions. *Indian J Agron* 51(1):17–20.

159. Mahrous NM, Safina SA, Hussien H, Taleb A, El-behlak SME (2015) Integrated use of organic, inorganic and bio fertilizers on yield and quality of two peanut (Arachis hypogaea L.) cultivars grown in a sandy saline soil. *Am J Agric Environ Sci* 15(6):1067–1074.

160. Singh S, Singh RN, Prasad J, Kumar B (2002) Effect of green manuring, FYM and biofertilizer in relation to fertilizer nitrogen on yield and major nutrient uptake by upland rice. *J Indian Soc Soil Sci* 50(3):313–314.

161. Mohiuddin MD, Das AK, Ghosh DC (2000) Growth and productivity of wheat as influenced by integrated use of chemical fertilizer, biofertilizer and growth regulator. *Indian J Plant Physiol* 5(4):334–338.

162. Kumar V (1994) Effect of Azotobacter chroococcum on Indian mustard grown in different soil environments. *Crop Res* 7(3):446–450.

163. Jadhav AS, Shaikh AA, Shinde AB, Harinarayana G (1990) Effects of growth hormones, biofertilizer and micronutrients on the yield of pearl millet. *J Maharashtra Agric Univ* 15(2):159–161.

164. Elsoni EM, Osman AG (2011) Effects of biofertilization on yield, physical characteristics and chemical composition of pigeon pea (Cajanus cajan L.). *Pakistan J Nutr* 10(10):978–981.

165. Bama ME, Ramakrishnan K (2010) Effects of combined inoculation of Azospirillum and AM fungi on the growth and yield of finger millet (Eleusine coracana Gaertn.) var. Co 12. *J Exp Sci* 1(8):10–11.

166. Singh RS, Yadav MK (2008) Effect of phosphorus and biofertilizers on growth, yield and nutrient uptake of long duration pigeonpea under rainfed condition. *J Food Legum* 21(1):46–48.

167. Singh AK, Singh RS (2012) Effect of phosphorus and bioinoculants on yield, nutrient uptake and economics of long duration pigeonpea (Cajanus cajan). *Indian J Agron* 57(3):265–269.

168. Reddy ASR, Babu JS, Reddy MCS, Khan M, Rao MM (2011) Integrated nutrient management in pigeon pea (Cajanus cajan). *Int J Appl Biol Pharm Technol* 2(2):467–470.

169. Maiti D, Toppo NN, Variar M (2011) Integration of crop rotation and arbuscular mycorrhiza (AM) inoculum application for enhancing AM activity to improve phosphorus nutrition and yield of upland rice (Oryza sativa L.). *Mycorrhiza* 21(8):659–667.

170. Velivelli SLS, et al. (2015) Identification of mVOCs from Andean rhizobacteria and field evaluation of bacterial and mycorrhizal inoculants on growth of potato in its center of origin. *Microb Ecol* 69(3):652–667.

171. Ceballos I, et al. (2013) The in vitro mass-produced model mycorrhizal fungus, Rhizophagus irregularis, significantly increases yields of the globally important food security crop cassava. *PLoS One* 8(8). doi:10.1371/journal.pone.0070633.
